# Critical Roles of Dual-Specificity Phosphatases in Neuronal Proteostasis and Neurological Diseases

**DOI:** 10.3390/ijms18091963

**Published:** 2017-09-13

**Authors:** Noopur Bhore, Bo-Jeng Wang, Yun-Wen Chen, Yung-Feng Liao

**Affiliations:** 1 Taiwan International Graduate Program in Interdisciplinary Neuroscience, National Yang-Ming University and Academia Sinica, Taipei 11529, Taiwan; noopurb11@gate.sinica.edu.tw; 2 Institute of Cellular and Organismic Biology, Academia Sinica, Taipei 11529, Taiwan; harry.wang@yahoo.com.tw (B.-J.W.); YWC120302@gate.sinica.edu.tw (Y.-W.C.)

**Keywords:** protein homeostasis, dual-specificity phosphatases, neuron, protein aggregates, heat shock response, oxidative stress, ER stress, autophagy

## Abstract

Protein homeostasis or proteostasis is a fundamental cellular property that encompasses the dynamic balancing of processes in the proteostasis network (PN). Such processes include protein synthesis, folding, and degradation in both non-stressed and stressful conditions. The role of the PN in neurodegenerative disease is well-documented, where it is known to respond to changes in protein folding states or toxic gain-of-function protein aggregation. Dual-specificity phosphatases have recently emerged as important participants in maintaining balance within the PN, acting through modulation of cellular signaling pathways that are involved in neurodegeneration. In this review, we will summarize recent findings describing the roles of dual-specificity phosphatases in neurodegeneration and offer perspectives on future therapeutic directions.

## 1. Introduction

Proteins are one of the most vital classes of molecules in the cell, carrying out myriad functions that range from enzymatic reactions to cell signaling. Precise functioning and durability of cellular proteins are maintained by the proteostasis network (PN), which governs the biosynthesis, folding and refolding, trafficking, aggregation, and degradation of proteins [1]. This network responds to intracellular alterations or the external microenvironment to sustain protein quality control and cellular functions under both normal and stressful conditions.

The PN comprises approximately over a thousand different factors that function both at an intracellular level and in a coordinated cell-nonautonomous manner [2]. The major categorical players in the PN, which directly and robustly affect the state of the proteome, are protein translational and post-translational machinery, trafficking machinery, molecular chaperones and the heat shock response (HSR), unfolded protein response (UPR), oxidative stress response (OxR), macroautophagy, and the ubiquitin-proteasome system (UPS). The auxiliary players, which exert major influence on these central processes, include cell signaling pathways, epigenetic modifiers, aging and metabolic factors [3,4]. These major and auxiliary players combine in each individual cell type to address the needs of the cell and tailor a cell-type specific PN. Because neurons exhibit unique morphology, lifespan and functional complexity, these cells rely heavily on the PN to operate seamlessly and provide an uninterrupted, robust network of functional protein units. Therefore, the involvement of each categorical player in the PN is currently under intense investigation in the context of toxic gain-of-function neurodegenerative diseases, such as Alzheimer’s disease (AD), Parkinson’s disease (PD), and Huntington’s disease (HD) [5].

Several major cell signaling pathways could influence the central pathways of PN. For example, the MAPK pathway has been implicated in governing protein aggregation, HSR, ER stress, and other stress-mediated responses in neuronal cells [6,7,8]. It is increasingly evident that post-translational modifications of cell signaling proteins represents a major control mechanism for proteostasis [9]. For example, Phosphorylation is one such modification, where the addition of one or more phosphate moieties is carried out by protein kinases and removal of phosphates is performed by protein phosphatases. Intriguingly, protein phosphatases comprise just 0.84% of the human proteome whereas protein kinases make up 2.39% [10], which may insinuate multiple downstream dephosphorylation targets for an individual phosphatase. The importance of balanced protein phosphorylation and dephosphorylation can be attested by the devastating effects of protein phosphorylation in stabilizing certain neurotoxic aggregates. Conversely, different phosphorylation events may ameliorate certain types of neurotoxicity [11], and dephosphorylation may be undesirable. Moreover, the interaction between PN and the physiological state of a certain protein assembly, is referred to as the quinary state of that protein [4]. It has been postulated that phosphorylation could influence this quinary state potentially by altering charge-charge interactions between interacting partners [12]. It provides another possible means by which phosphorylation, and further, dephosphorylation could influence proteostasis.

**Figure 1:**
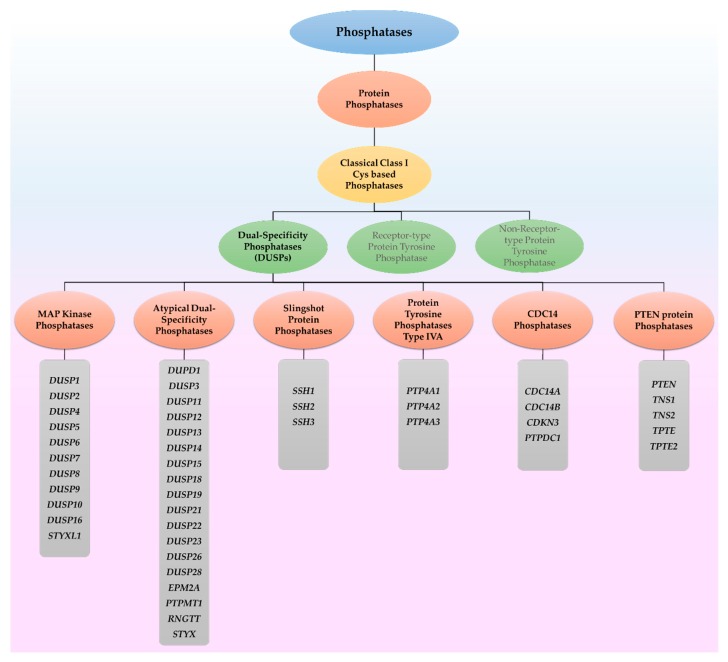
The schematic classifications of human dual-specificity phosphatases. Phosphatases are classified into seven gene families, of which Protein Phosphatases are one. They are further categorized into five groups, which includes Class I classical Cys-based Phosphatases. This group is then subdivided into dual-specificity phosphatases, Receptor-type Protein Tyrosine Phosphatases, and Non-receptor-type Protein Tyrosine Phosphatases. Dual-specificity Phosphatases are categorized by six subfamilies: (i) Mitogen-activated Protein Kinase Phosphatases (MKP); (ii) Atypical DUSPs; (iii) Slingshot Protein Phosphatases; (iv) Protein Tyrosine Phosphatases type IVA; (v) CDC14 Phosphatases and (vi) PTEN Protein Phosphatases. Members of each subfamily are as listed in the figure. Data are adapted from the HUGO Gene Nomenclature Committee at the European Bioinformatics Institute, http://www.genenames.org/.

**Figure 2:**
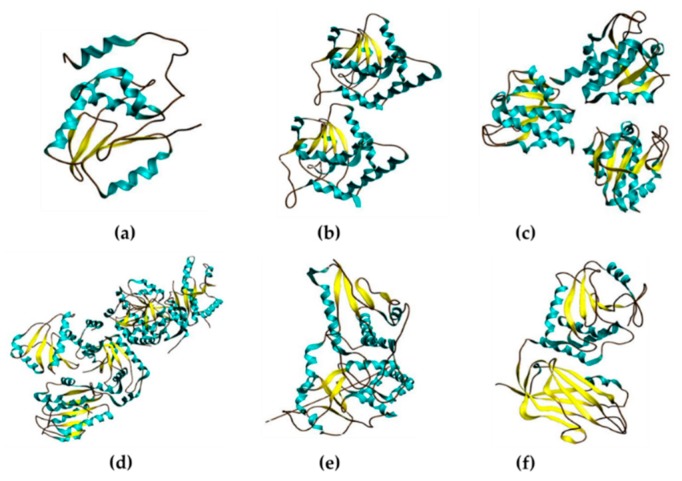

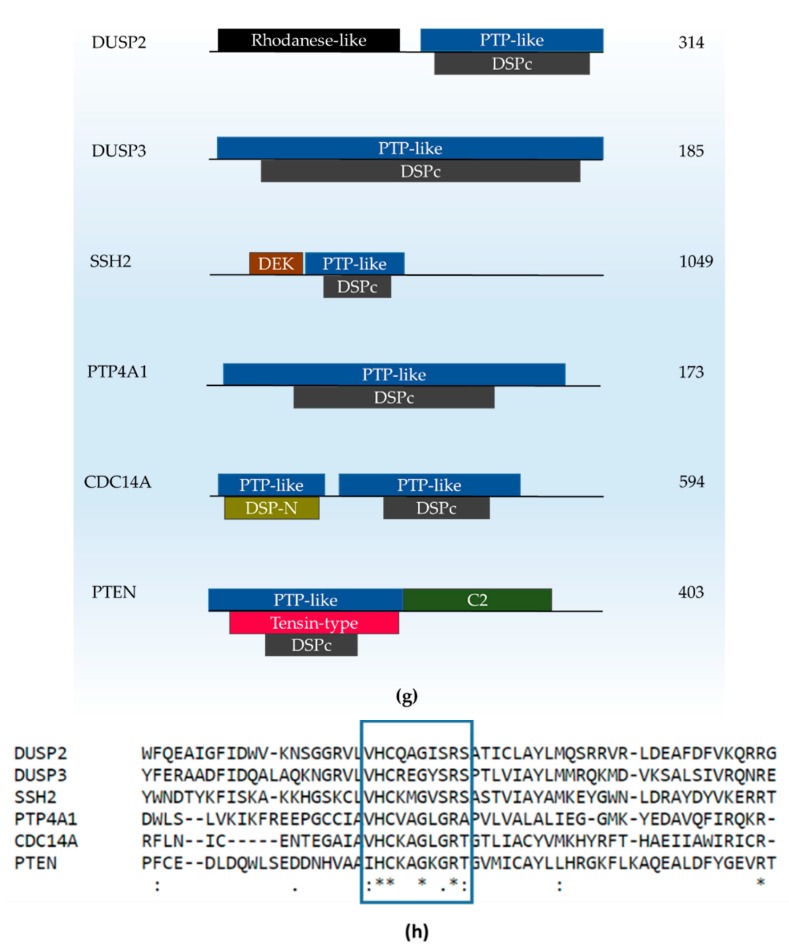
Structural features of typical members from each DUSP subfamily. (**a**–**f**) Molecular representations of typical member from each DUSP subfamily using data available from Protein Data Bank (PDB) and redrawn using Avogadro: an open-source molecular builder and visualization tool, version 1.XX, http://avogadro.cc/. Cyan color on the structure indicates helix, yellow color indicates sheet, and brown color represents loop structures; (**a**) Image of 1M3G represents DUSP2 structure [20] of the MKP subfamily; (**b**) Image of 3F81 represents DUSP3 structure [21] of the atypical-DUSP subfamily; (**c**) Image of 2NT2 represents SSH2 structure [22] of the slingshot phosphatase subfamily; (**d**) Image of 1XM2 represents PTP4A1 structure [23] of the PTP4A phosphatase subfamily; (**e**) Image of 1OHC represents CDC14A structure [24] of the CDC14 phosphatase subfamily; (**f**) Image of 1D5R represents PTEN structure [25] of the PTEN phosphatase subfamily; (**g**) Domain representation of typical member of each DUSP subfamily: DUSP2, DUSP3, SSH2, PTP4A1, CDC14A and PTEN created from data available on InterPro [26] (not drawn-to-scale). Abbreviations of domains listed in the figure include, PTP-like: Protein tyrosine phosphatase-like; DSPc: Dual-specificity phosphatase, catalytic; DSP-N: Dual-specificity phosphatase, N-terminal. Numbers on the right side indicate amino acid length. It should be noted that variations exist in individual members from each subfamily in presence/absence of protein domains and taken into consideration. For further information on protein domains of an individual DUSP, please refer to Table 1 and [27]; (**h**) Multiple sequence alignment of typical members of each DUSP subfamily: DUSP2, DUSP3, SSH2, PTP4A1, CDC14A and PTEN. Amino acid sequences were obtained from UniProt [28], and aligned using Clustal Omega at EMBL-EBI [29,30]. Blue box indicates the conserved catalytic DUSP motif (V)-HC-XX-X-XX-R-(S/T), where X represents any amino acid; (:) indicates conservation between groups of strongly similar properties; (*) indicates a conserved residue; (.) indicates conservation between groups of weakly similar properties.

Dual-specificity phosphatases (DUSPs) are Class I classical cysteine-based protein phosphatases that have the dual ability to dephosphorylate phospho-serine/threonine and phospho-tyrosine residues. The first evidence of dual-specific phosphatase activity was reported by Guan et al. in 1991 for vaccinia virus VH1 phosphatase. There are now 44 different human DUSPs that have been identified and grouped into six subfamilies: (i) Mitogen-activated Protein Kinase Phosphatases (MKPs); (ii) Atypical DUSPs; (iii) Slingshot Protein Phosphatases; (iv) Protein Tyrosine Phosphatases type IVA; (v) CDC14 Phosphatases and (vi) PTEN Protein Phosphatases, as listed by the HUGO Gene Nomenclature Committee. Figure 1 illustrates the sub-classifications for the different members of the DUSP family, whereas Figure 2 delineates the structural features of representative members from each DUSP subfamily. The alternative names of the DUSP members are listed in Appendix A. The classical DUSPs, or MKPs, are involved in dephosphorylating mitogen or stress-activated ERK, JNK and p38 kinases. The substrates of atypical DUSPs are varied, and include: ERK, JNK, p38, STAT, AKT, and PI(5)P. Some targets of CDC14 family include proteins like ERK3, p53, RN-tre, CDK2, PLK1, while those of PTP14 family includes ezrin, EF-2, ATF-7, p53, and KIT. Substrates of Slingshot protein phosphatases include ADF, cofilin and LIMK1 (HGNC), and those of the PTEN protein phosphatase family include PIP3, PP1α, and AKT [13,14,15,16,17]. From the above examples, it is clear that DUSPs regulate various essential cell signaling pathways. Furthermore, the importance of DUSPs is rapidly gaining ground based on studies in neurodegenerative disease models. For example, DUSP26 has been shown to stimulate Aβ production during hypoxia, while DUSP1 expression is upregulated in PD, and was shown to be neuroprotective against mutant Huntingtin [6,18,19]. DUSPs may therefore be considered as candidate therapeutic targets with the potential for manipulating disease microenvironments.

The question then arises—how do DUSPs influence proteostasis? In this review, we will discuss existing evidence that DUSPs function to surveil the PN, primarily, by regulating cell signaling and thereby affecting a few of the central PN pathways. We will then provide a unifying model on how DUSPs regulate these central pathways which come together during neuronal proteostasis. Lastly, we will offer perspectives on modulating DUSPs for therapeutic application.

## 2. Mechanisms by Which DUSPs May Affect Neuronal Proteostasis

DUSPs bear a conserved catalytic motif H-C-X-X-X-X-X-R-(S/T), where X could be any amino acid. While this class of molecules regulates many proteins by serine/threonine and tyrosine dephosphorylation, DUSPs are themselves regulated by transcription, post-translational modifications and catalytic modulation [71]. Intriguingly, several DUSPs have been linked to various neurological disorders, including several neurodegenerative diseases, as indicated in Table 1. Some DUSPs which do not have a clear role in neurological diseases have otherwise been associated with neuron or oligodendrocyte development, and thus may potentially play as yet unidentified roles in neuronal dysfunction. Additionally, two DUSPs have appeared in genome-wide association studies (GWAS) of neuronal disorders and await further confirmation, and only a few remain unassociated with neurological diseases. Here, we will overview some means by which DUSPs may participate in neuronal proteostasis.

### 2.1. DUSPs Act through Mitogen-Activated and Stress-Activated Protein Kinases

Mitogen- and stress-activated protein kinases (MAPK/SAPKs; hereafter referred as MAPKs) are one of the chief cell signaling pathways that phosphorylate proteins on Ser/Thr/Tyr residues to induce responses in a cascade of downstream effectors. The MAPKs are involved in cell signaling, cell cycle, chromatin remodeling, cell fate determination, neuronal plasticity, learning and memory, and apoptosis [72,73]. In particular, the extracellular signal-regulated kinases (ERK) have been implicated in oxidative stress, stroke, seizure, Lewy body immunoreactivity, tau phosphorylation, and excitoxicity [74,75]. Similarly, the c-Jun N-terminal kinase (JNK) signaling is involved in tau-induced neurotoxicity, modulating amyloid-β levels, excitotoxicity, ischemia, neuroinflammation, and oxidative stress [76]. The p38 signaling pathway regulates tau phosphorylation, inflammatory response, focal cerebral ischemia, excitoxicity, α-synuclein mediated activation, and colocalization with amyloid-β [77]. Moreover, MAPKs often regulate the transcription of downstream *DUSP* genes, thereby creating a negative feedback loop [78].

MKPs interact with MAPKs via several sites in addition to the MAPK-binding domain that defines the subfamily. This complex interaction may allow some DUSPs to exhibit preferential dephosphorylation of certain MAPKs compared to others. For example, DUSP1 more readily dephosphorylates JNK and p38, than ERK. The differences in substrate specificity among classical DUSPs/MKPs are attributed to various interaction sites, particularly, in the Rhodanese (containing MAPK-binding sites) and catalytic domains [13]. The atypical DUSPs, on the other hand, have varied dephosphorylation substrates which also include the MAPKs, despite the lack of a specific MAPK binding motif in atypical DUSPs [13]. There is no information currently available on whether DUSP subfamilies other than MKPs and atypical DUSPs can dephosphorylate MAPKs. However, like atypical DUSPs, the other subfamilies lack a defined MAPK-binding domain [27], (Table 1), suggesting that the interactions may be variable between individual proteins.

### 2.2. DUSPs Act through Other Mechanisms Based on Their Unique Functional Domains

All DUSP subfamilies have unique features in substrate docking motifs, conformation or specific domains which can recognize different substrates. Some examples of these unique features include slingshot phosphatase domains of the Slingshot subfamily, tensin-type phosphatase domain of the PTEN subfamily, a Pro residue in the active site of CDC14B, and shallow active site cleft and hydrophobic residues in the signature motif of the PTP4A subfamily. On the basis of these and other unique features, various DUSPs are capable of functioning as mRNA-capping enzymes, scaffolding phosphatases and scaffolding pseudophosphatases, mitochondrial phosphatases, or dual-specificity protein-and-glucan phosphatases. A concise description of the various domains in different DUSP family members is provided in Table 1, and excellent, detailed reviews on the various domains and features of DUSPs have been published previously [14,71]. Evidence for these alternative mechanisms in regulation of neuronal proteostasis are not aplenty, leaving a wide scope for potential future investigations.

## 3. DUSPs in Protein Aggregation Diseases

The relevance of protein phosphorylation as a modifier of proteostasis in certain aggregation-prone neuronal proteins has been previously described. For example, hyperphosphorylation of the neuronal tau protein at Ser199, Ser202, and Thr205 is recognized as a key event that leads to the formation of neurofibrillary tangles and synaptic loss in various tauopathies [11]. Evidence also point to the involvement of α-synuclein phosphorylation at sites Ser87, Ser129, Tyr125, Tyr133, and Tyr136 in PD etiology. Phosphorylation of amyloid-β at Ser26 leads to its stabilization and subsequent increase in its neurotoxicity, and moreover, phosphorylation of TDP-43 at Ser379, Ser403, Ser404, Ser409, and Ser410 also boosts aggregate formation [79,80].

On the other hand, phosphorylation of certain proteins or blocking certain phosphatases can also be helpful for maintaining neuronal health. For example, phosphatases, PP2B and STEP, have been implicated in promoting the pathogenesis of AD [81]. Furthermore, some reports suggest that eIF2α dephosphorylation is important in proteinopathies [82]. Several reports have indicated that some phosphorylation events may decrease the levels of toxic protein assemblies and even promote their degradation [11,80]. Perhaps the strongest example for the beneficial effects of phosphorylation has been reported for huntingtin, whose phosphorylation at Ser13, Ser16, or Ser421 could promote its clearance by the ubiquitin-proteasome system [80]. Furthermore, phosphorylation at Thr3 of huntingtin can reduce neurotoxicity by forming microscopic aggregates that offset HD pathogenesis [80]. Whether the effects of phosphorylation are protective or toxic, all of these examples nevertheless underscore the crucial impact of dephosphorylation as the diametrically opposite regulatory process. It is interesting to note that phosphorylation occurs at Ser residues 95% of the time, followed by Thr (4%) and Tyr (1%) [10], thus placing dual-specificity phosphatases at an advantage among other dephosphorylating moieties. In this section, we will define the possible means by which DUSPs could participate in the protein aggregation response.

Several DUSPs can regulate MAPKs or related proteins through dephosphorylation. For example, DUSP1 has been shown to dephosphorylate JNK and p38 kinases in an HD model and its expression is increased in the 6-hydroxydopamine (6-OHDA) rat model of PD, suggesting that DUSP may be neuroprotective in both diseases [19]. BDNF-induced DUSP1 can dephosphorylate JNK and affect axonal branching [83]. The levels of both DUSP1 and DUSP6 are decreased in cases of familial amyloidotic polyneuropathy, and the levels of phospho-ERK are elevated leading to subsequent cytotoxicity [84]. DUSP6 knockdown can increase the level of phospho-ERK to promote high levels of tau phosphorylation. Interestingly, the protein level of DUSP6 was found to be decreased in AD brain lysates [85]. DUSP26 has been shown to regulate amyloid-precursor protein (APP) for amyloid-β production by inducing JNK phosphorylation [6]. Additionally, DUSP16 can dephosphorylate JNK3 that is bound to β-arrestin 2 in COS-7 cells, which may also occur in neurons [86,87]. Although no significant upregulation of JNK phosphorylation was observed in sensory (dorsal root ganglia) neurons isolated from *DUSP16* knock-out mice, we suppose the discrepancy could be due to the different systems used in each study and also the absence of JNK activator, ASK1, in the knock-out mouse model [39].

Apart from MAPKs, there are various other signaling targets that are modulated by DUSPs. p53 is associated with neurodegenerative diseases like AD, PD, and HD, where it participates in processes that regulate or respond to apoptosis, mitochondrial dysfunction, neuronal injury and possibly, protein misfolding [88]. The *DUSP16* knock-out has been shown to enhance phosphorylation of p53 at Ser15 in sensory neurons upon trophic factor withdrawal [39]. Analogously, DUSP26 can also dephosphorylate p53 at Ser20 and Ser37, thus suggesting a role for DUSPs in regulating p53-mediated pathways [89]. DUSP22 has been shown to be induced by the pro-inflammatory cytokine interleukin-6 (IL-6) and could dephosphorylate STAT3 in hepatoma cells, creating a feedback loop for the IL-6/STAT3 signaling [90]. Curiously enough, IL-6 can prompt several downstream responses such as upregulation of cdk5/p53 complex and phosphorylation of STAT3 and ERK, all of which integrate to hyperphosphorylate tau protein [90]. Whether DUSP22-mediated regulation of IL-6 has any implications in tauopathies may be an interesting topic for study, since it has already been shown that DUSP22 can influence tau phosphorylation via a protein kinase A-dependent pathway [49].

Actin depolymerizing factor (ADF)/cofilin are actin binding proteins which regulate the dynamics of actin polymerization during axonal transport and neurodevelopment [91]. Slingshot phosphatases can dephosphorylate and thus activate cofilin. Under stressful conditions, activated cofilin has a propensity to aggregate with ADP-actin, forming cofilin rods that hinder vesicular transportation and promote neurite atrophy. Consequently, cofilin rods have been associated with glutamate excitoxicity, oxidative stress, amyloid-β, neuropil threads, huntingtin, and ischemia [92]. Blocking slingshot-mediated dephosphorylation can at least partially prevent induction of cofilin rods [93]. Recently, it was demonstrated that cofilin can associate with the cellular form of prion protein (PrP^C^) in sporadic Creutzfeldt-Jakob disease subtypes and higher levels of SSH1 could be detected in disease samples. This study creditably underscores the cofilin-SSH1 interaction as a contributor of neurodegeneration [94]. Further, PTEN is a lipid and protein phosphatase that inhibits PI3/AKT signaling and inhibiting PTEN has neuroprotective effects in an AD mouse model, amyloid-β toxicity, a PD model, and lab models of spinal muscular atrophy [95]. PTEN inhibition has also been shown to reduce apoptosis and counteract ER-stress related proteins in an AD mouse model [96]. In contrast, however, PTEN overexpression seems to be neuroprotective in tauopathies [97]. In conclusion, DUSPs should be easily recognized as critical regulators of protein aggregation, which occurs mainly by manipulating phosphorylated proteins.

## 4. DUSPs in the Heat Shock Response Pathway

The heat shock response (HSR) is a conserved proteostasis pathway that restores proper conformation of proteins which become unfolded or aggregated under physiological or stressful conditions. The general mechanism of the HSR involves (a) induction of various signaling cascades in response to stress; (b) activation of the heat shock transcription factors (HSFs), such as the activation of HSF1 by dissociation with its binding partner Hsp90; (c) transcriptional activation of various heat shock proteins (Hsps) by HSFs and (d) refolding of proteins or ubiquitination for degradation [98]. Different kinds of stresses including exposure to high temperatures, heavy metals, or oxidative stress can induce the expression of similar sets of Hsps. There are various classes of heat shock proteins, however, the molecular chaperones are particularly important in neuroprotection and include such proteins as Hsp40, Hsp60, Hsp70, Hsp90, Hsp100 and small Hsp families [99,100]. The involvement of HSR in neurodegenerative diseases may be illustrated by several examples—for one, Hsp70 promotes a decrease in α-synuclein levels in dopaminergic neurons. Additionally, expression of Hsp70, Hsp60 and Hsp40 protects against amyloid-β induced toxicity. Furthermore, Hsp27 protects against superoxide dismustase-1 induced toxicity in an amyotrophic lateral sclerosis (ALS) disease model [101]. Besides the involvement of Hsps in neuroprotection, all three previously mentioned MAPK pathways—ERK, JNK, and p38—are also induced in response to HSR-inducing stressors [7].

From previous works, we may see that several DUSPs are modulated in response to heat shock, and some may also interact directly with Hsps. Although this may not have been necessarily shown in neuronal systems, the HSR pathway is a highly conserved one, and we suspect that some of the mechanistic associations between DUSPs and HSR signaling might also exist in neurons. For instance in Cos-7 cells, the stress-inducible Hsp72 could prevent heat shock-mediated aggregation of DUSP1 and DUSP6, inhibit the activation of ERK signaling, and as a possible consequence, may decrease the survival of stress-damaged cells [102]. The expression of these DUSPs and stress-inducible Hsp72 in neuronal cells suggest that this mechanism may also be at play in neurodegeneration. However, it should be noted that Hsp72 is endogenously expressed only in certain neuronal cell lines [103]. Furthermore, the mouse ortholog of DUSP8 (M3/6) is susceptible to heat shock and tends to aggregate as well, stimulating a concomitant rise in phospho-JNK levels [104]. Polyglutamine stress can also elicit a similar response, though appropriately, Hsp70 expression restricts M3/6 aggregation as well as JNK activation in this model [105].

Stress-mediated ERK activation can induce Hsp70 in neuronal cells. Upon persistent ERK activation, vaccinia-related kinase 3 (VRK3) promotes nuclear localization of Hsp70, which then interacts with DUSP3 to suppress elevated ERK activation. This suggests a route by which dephosphorylation may suppress detrimental ERK levels in neuronal cells [106]. Another phosphatase, DUSP26, can interact with and dephosphorylate the phospho-ERK-activated heat shock transcription factor Hsf4b [107]. Again, all these proteins are expressed in brain regions and have been shown to interact similarly. In non-neuronal cells, it was found that DUSP12 interacts with Hsp70, accumulates in perinuclear region, and protects the cells in response to heat shock [108]. Whether this can hold true in neurons remains to be seen. In contrast, Cdc14 dephosphorylates yeast Hsp90 on a residue that is conserved in the human isoform, but whether this action may occur in neurons is undetermined [109]. Hsp90 inhibition is known to be beneficial for cell survival, although not on a long-term basis. Additionally, it is known that DUSP5 and SSH-1 are likewise susceptible to heat shock as they become inactivated, but whether this is also true in neurons is yet again undetermined [110,111]. In the above examples, we observe a pattern where DUSPs and their dephosphorylation substrates are affected in response to heat shock, and thus may affect the proteostasis signaling repertoire of the afflicted cells. However, modulation of the remaining DUSPs in the context of HSR remains to be probed.

## 5. DUSPs in Oxidative Stress Response

Another distinct pathway acts to combat oxidative stress in the cell. Oxidative stress is essentially the disruption of harmony between reactive oxygen species (ROS) and antioxidant mechanisms. Examples of ROS in the cellular environment include free radicals such as hydroxyl species (OH), superoxide anion (O_2_^−^), and peroxynitrite (ONOO^−^) [112]. Neurodegenerative diseases like AD, PD, and HD include a component of oxidative stress that may be derived from excess ROS production, loss of antioxidant defenses, toxic protein aggregate accumulation, inflammation, mitochondrial dysfunction, or other sources [113]. In general, the protein tyrosine phosphatases are susceptible to oxidative stress at the catalytic cysteine residue, but the presence of an additional Cys residue near the active site of certain DUSPs renders them comparatively less prone to oxidative damage by forming a disulfide bond with the catalytic cysteine. In evidence of this, DUSP4, DUSP13b isoform, DUSP16, and DUSP28 were shown to be capable of recovering more than 70% of their activity after oxidation in one particular study [114]. Since most DUSPs are expected to recover their activity in oxidative conditions, in this segment we will describe how DUSPs may coordinate with the various modulators of oxidative stress response (OxR) to play a role in this aspect of proteostasis.

ERKs are phosphorylated in a cell-type specific manner during oxidative stress, and increased expression is often observed in brain regions that ultimately undergo cell death. DUSPs are important negative regulators of ERK phosphorylation, which is under strict spatiotemporal control by multiple factors, and conversely, ERKs can phosphorylate, and hence activate, downstream DUSPs to generate a negative feedback loop. Thus, it may be suggested that DUSPs may exhibit a critical neuroprotective role of dephosphorylating ERK during conditions of elevated oxidative stress [115,116]. Further, DUSP1 induction was observed in a neuroblastoma cell line under conditions of hypoxia/reoxygenation, and this induction was involved in the downregulation of pro-apoptotic genes and neuronal death [117]. In addition, ROS-induced DNA damage is sensed by PARP-1 whose activity is known to be increased in neurodegenerative diseases like AD and PD. PARP-1 inhibition can exert therapeutic effects partly by increasing DUSP-1 levels, which is followed by reduction in JNK and p38 phosphorylation, as seen in non-neuronal cells [118,119]. Whether the same results can be repeated in neurons remains to be seen. Another interesting example is that of M3/6, which changes its substrate preference from JNK1β and JNK2α to JNK1α and JNK3 isoforms after arsenite-induced oxidative stress. This shift in substrate preference could then affect isoform-specific downstream signaling modules, an observation that is also yet to be replicated in neurons [120].

Furthermore, atypical DUSP PTPMT1 is induced in response to hypoxia by hypoxia-inducible factor HIF-2α in erythroid leukemia cells, and its inhibition induces apoptosis [121]. Whether HIF proteins induce PTPMT1 in non-cancerous neurons under hypoxic conditions is yet another open question, especially since *PTPMT1* single-nucleotide polymorphisms are possibly associated with AD [122]. In one study, inhibition of PTEN was shown to protect neuroblastoma cells against toxicity, oxidative stress, and apoptosis induced by amyloid-β_25–35_ [123]. Oxidative stress can also lead to inhibition of PTP4A1 phosphatase activity in photoreceptor cell models indicating a potential role in stress management [124]. One of the *Drosophila* DUSPs, Puckered, was phosphorylated upon induction of oxidative stress and then dephosphorylated stress-induced JNK [125]. On the other hand, slingshot phosphatase, SSH1, was activated by ROS formation and it in turn activated the cofilin proteins, leading to the formation of cofilin rods which are responsible for neurite atrophy [126]. The involvement of DUSPs during oxidative stress response in neurons is clearly important, and hence, investigation of potential roles for DUSPs in regulating oxidative stress response can offer new avenues for the development of novel therapeutics.

## 6. DUSPs in Endoplasmic Reticulum Stress, Autophagy and Apoptosis

### 6.1. Endoplasmic Reticulum Stress

The endoplasmic reticulum (ER) governs synthesis, folding, and transportation of proteins in a cell. Environmental or physiological stressors such as viruses or gene mutations that cause protein misfolding can overwhelm the quality control systems in the ER, and trigger the ER stress response. An adaptive ER stress response, called the unfolded protein response (UPR), resolves imbalances in protein folding and maturation, accumulation of misfolded proteins or blockades in protein trafficking. The UPR includes PERK, IRE-1α and ATF-6 signaling pathways which induce responses like translation inhibition, antioxidant defenses, ER-associated protein degradation (ERAD), and autophagy. When the UPR can no longer manage ER stress due to accumulation of misfolded proteins or overexposure to other stressors, the distressed cell may commit to programmed cell death (apoptosis), in order to minimize adverse effects on the tissue. Apoptosis induction after ER stress relies on CHOP, IP3R, RYR, JNK, and ASK1 signaling pathways to activate proapoptotic proteins and eventually caspase cleavage [127,128].

Based on previous literature, it is known that the MAPKs act in concert with the ER stress response. ERK signaling may promote cell survival upon the induction of ER stress, possibly, by activating anti-apoptotic factors like BCL-2 and BCL-XL and deactivating pro-apoptotic factors like BIM and PUMA. At least some studies suggest that IRE-1α may activate ERK signaling under stressful conditions, and both IRE-1α and CHOP are known to activate JNK signaling. The JNK pathway is involved in the upregulation pro-apoptotic factors like phosphorylated BIM and BCL2. Another effect of JNK signaling may be to promote cell survival by phosphorylating BCL2, stimulating its dissociation from Beclin1, and thus leading to the induction of autophagy. p38 also acts as a pro-apoptotic signaling molecule under stressful conditions. This kinase can promote cell cycle arrest by activating MK-2, and cell death by phosphorylating proteins like BIM and p53. Moreover, it can also activate ATF6 and CHOP signaling [8]. Hence, depending on the set of substrates that are phosphorylated by different MAPK proteins, different responses may be evoked within the context of ER stress signaling. Therefore, DUSPs that deactivate MAPK signaling certainly have a role to play in fine-tuning the MAPK signaling cascades within ER stress response signaling.

There has been little research on the connections between DUSPs and ER stress in neurodegenerative disease, making it a fresh field to explore. The localization of DUSPs around the ER is likely a deciding factor in whether they participate in ER stress response. However, a few reports do offer flickering insights into the role of DUSPs in this context. DUSP1 has a role in activating BCL2 and caspases, and decreasing the neuroprotective protein CEBP/β during ischemic injury [129]. Further, one study has shown that inactive PERK may indirectly affect the nuclear transportation of PTEN and sequester it to the cytoplasm [130]. Since the consequence of activating PTEN is known to be the inactivation of PI3K/AKT signaling, under ER stress conditions, AKT activation may be expected to be reduced. However, given the ambiguous role of PTEN in neurodegeneration, we suspect the consequences of PERK inhibition on PTEN/PI3K/AKT axis could be complex and either result in protection or toxicity depending on the proteopathy model being studied.

### 6.2. Autophagy

Autophagy is another well-known process that participates in cellular stress responses to affect proteostasis. The general mechanism involves (i) the initiation of autophagy at phagophore assembly site (PAS); (ii) nucleation of the phagophore membrane that engulfs misfolded proteins and damaged organelles; (iii) membrane elongation leading to the genesis of an autophagosome and (iv) fusion with a lysosome to form an autophagolysosome structure, which degrades the engulfed contents and allows them to be recycled. Several proteins are well-known primary contributors to autophagic progression, including (a) mTORC1 dependent–ULK complex and mTORC1-independent AKT and EGFR signaling during the formation of PAS; (b) the Beclin1–Vps34 complex during nucleation; (c) ATG12–ATG5–ATG16L and LC3–phosphatidylethanolamine (PE) complexes, which contribute to phagophore expansion; (d) autophagy receptors and adaptor proteins that tether target proteins to be degraded and (e) mTORC1, which helps in terminating autophagy [131,132].

Because the MAPK pathways can crosstalk with autophagy pathways, MAPKs are important regulators that may influence the outcome of the autophagic progression. For example, ERK signaling can activate autophagy in neurons in response to neurotoxins and has been associated with non-apoptotic neuronal death which is suspected to be autophagic in nature [133]. JNK signaling also activates autophagy by regulating the transcription of *Atg* genes, and phosphorylates BCL-2, causing its dissociation from Beclin1 to promote autophagy [134]. p38 signaling, on the other hand, has a cell-type dependent effect on activation and inhibition of autophagy [135]. Autophagy modulation or dysfunction has been noted in several neurodegenerative diseases, including AD, PD, HD, ALS, and DUSP EPM2A-induced Lafora disease [136].

DUSP1 provides an illustrative example of the effects of phosphatase mediated regulation of autophagy. Knockdown of *DUSP1* leads to induction of autophagy in ERK-dependent manner as observed in ovarian cancer cells. *DUSP1* knockdown probably mediates this effect via reduced dephosphorylation of ULK and increased LC3II formation which then results in autophagosome formation and maturation [137]. DUSP1 may also dephosphorylate the scaffolding protein, JIP1, to maintain retrograde transport of autophagosomes in axons, thus allowing them to mature and help in protein clearance [138]. In addition to DUSP1, the yeast homolog of DUSP12, YVH1, was shown to aid in the formation of the PAS structure after TORC1 inactivation which triggers the initiation of autophagy [139]. In another instance, PTEN phosphorylation and nuclear translocation led to the induction of autophagy in cancer cell lines that experienced topotecan-induced DNA damage [140]. Moreover, Laforin also positively increased autophagy by increasing the levels of LC3II [141].

### 6.3. Apoptosis

Apoptosis is the process of programmed cell death, in which caspases are activated through either the extrinsic or intrinsic activation pathways. The extrinsic pathway is initiated by the binding of death receptors to their ligands, which then activates caspase 8 and finally, the downstream effector caspases—caspase-3 and caspase-7. The intrinsic pathway is triggered by intracellular stimuli such as DNA damage or ER stress, which induce mitochondrial outer membrane permeability (MOMP) and the release of cytochrome C to the cytosol. Once in the cytosol, cytochrome c activates the apoptosome, including caspase-9, and then subsequently activates effector caspase-3 and caspase-7. Ultimately, effector caspase activation is irreversible and leads to cell death [142,143]. The MAPK signaling pathways have been implicated in mediating signals that initiate apoptosis in various neurodegenerative diseases, such as AD, PD and ALS [143,144]. Naturally, some DUSPs have been shown to be involved in regulating the MAPK proteins to influence apoptosis. As an example, DUSP1 can dephosphorylate JNK proteins and hence play a role in downregulating apoptosis upon growth factor withdrawal in cervical ganglion neurons [145]. DUSP13a, on the other hand, can induce apoptosis through ASK1/caspase-3 or casapase-9 signaling axis [45]. Meanwhile, STYXL1 is a mitochondrial phosphatase that opposes activation of the intrinsic apoptotic pathway by modulating MOMP and may have potential to enhance cell survival [146].

Thus, we find that various DUSPs play disparate roles in regulating the ER stress response, autophagy and apoptosis. However, it remains to be investigated if other members of the DUSP family have any potential roles in these branches of proteostasis.

## 7. Discussion

As critical regulators of dephosphorylation, DUSPs are recognized to be centrally involved in a variety of cellular and biochemical processes. They are commonly encountered points of control for MAPK signaling in numerous biomedical contexts. Emerging reports of DUSP involvement in other crucial signaling pathways, such as PI3/AKT or STAT signaling, corroborates their importance in the cell. This protein family is well-known to be involved in many cellular functions such as cell cycle regulation, proliferation, and differentiation. Moreover, some DUSPs can also function as scaffolding proteins, mRNA capping enzymes and glucan-binding moieties [71]. Interestingly, previous work has also demonstrated the involvement of DUSPs in tissue or organism-wide responses, such as immune response and tumor suppression [78]. Moreover, the subcellular or tissue specific localization of a DUSP may have influences on its activity. With so much diversity in functional targets, it is clear that DUSPs are important components of the cellular machinery.

**Figure 3:**
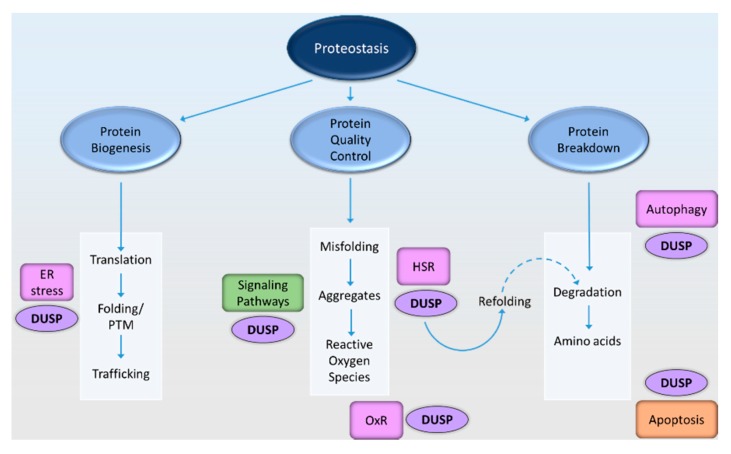
A proposed working model showing the involvement of DUSPs in pathways of proteostasis that contribute to neurodegeneration. A simplified version of proteostasis is represented under three central themes—protein biogenesis, protein quality control processes, and protein degradation. In this article, we highlight the role of DUSPs in protein quality control and breakdown, with respect to neurological disorders. Protein translation, folding, and transport occur largely within the endoplasmic reticulum (ER). An increased load of misfolded proteins in the ER evokes the ER stress response, and several DUSPs have been shown to participate in this pathway of proteostasis. Next, protein aggregates are the by-products of accumulated misfolded proteins and represent the hallmarks of many neurodegenerative diseases. DUSPs participate in phosphorylation-dependent modulation of protein aggregation mostly by regulating MAPK and related signaling pathways. Reactive oxygen species (ROS) production is often triggered in response to protein aggregates and results in oxidative stress. DUSPs participate in the oxidative stress response (OxR), and may have protective or aggravating roles, depending on the phosphatase. Further, DUSPs have a confirmed involvement in the heat shock response (HSR) pathway by either self-modulation or by direct interaction with the heat shock proteins/molecular chaperones. Heat shock proteins assist misfolded and aggregated proteins to refold and attain their native conformation. Proteins which fail to refold even after assistance from the heat shock response pathway, may then be degraded (indicated by dotted arrow). Finally, autophagy is the major degradation route for toxic-protein aggregates, and is known to be influenced by some DUSPs. When individual cells become overwhelmed by proteotoxic stress, they may enter apoptosis. The initiation of the apoptotic cascade is also known to be influenced by certain DUSPs.

The involvement of DUSPs in distinct individual pathways of neuronal proteostasis have been described and presented herein, however, an integrated view of how DUSPs orchestrate multiple proteostasis pathways is still emerging. In this review, we have summarized the known roles of DUSPs that may influence progression of protein aggregation diseases. In this context, regulation of cell signaling-mediated phosphorylation events may modulate proteotoxicity by influencing a variety of proteostatic processes. Two of the most important defensive processes in neurodegenerative disease are the stress responses to heat shock and the oxidative stress, and accumulating evidence shows that DUSPs are centrally involved in regulating these responses. In addition, we describe the initial findings with regards to DUSP-mediated regulation of ER stress, autophagy, and apoptosis. An illustrated working model recounts the involvement of DUSPs in neuronal proteostasis as shown in Figure 3.

Based on the widespread regulatory activities of DUSPs it may be prudent to consider them as potential therapeutic targets for neurodegenerative diseases. DUSPs exhibit high diversity in their downstream targets, which is an important consideration that may be exploited in the development of therapeutic strategies. DUSPs all bear a protein-tyrosine phosphatase (PTP) domain, however, the active site pocket of DUSPs is shallower than most PTPs [14]. This allows for some level of promiscuity in target recognition and perhaps can provide a reason for their dual-specificity towards phospho-Ser/Thr and phospho-Tyr. Moreover, several DUSPs have their own characteristic domains or motifs, such as the SH2 domain or PTB/PI domain (Table 1), which can also be considered as sites to potentially modulate DUSP activity. We provide an exhaustive list of inhibitors and activators of DUSPs which have been validated in previous literature in Table 2. Several of these chemical compounds are already available as commercial pharmacological drugs, whereas others are merely non-specific inhibitors of phosphatase activity. Indeed, sodium orthovanadate is a classical phosphatase activity inhibitor and can inhibit most DUSP phosphatase activities [147]. To our knowledge, there are only a few DUSP activators that are known. Besides small molecule inhibitors and activators, DUSP modulation may be achieved by physiological means, including transcription, epigenetic or post-translational modifications, subcellular localization, and manipulation of DUSP catalytic activities of DUSPs by upstream modulators.

Rightfully, DUSP manipulation has been suggested as a therapeutic strategy in several diseases apart from neurodegeneration such as in cancer, arthritis, diabetes, ischemia/neuronal injury, and cardiomyopathy among others [148]. In addition, there are several reports indicating the possible association of DUSPs with neurological conditions other than those mentioned in this article. For instance, the individual association of DUSP2, DUSP 4, DUSP 6, DUSP 8, DUSP 11, DUSP 13, DUSP 24 has been reported with ataxin-1, which is the causative protein of spinocerebellar ataxia type 1 [149]. The splicing abnormalities of DUSP22 were shown to occur in spinal muscular atrophy motor neurons [150]. *STYXL1* has recently been suggested as a candidate gene involved in intellectual disability and seizures [151]. A few DUSPs appeared to be dysregulated in major depressive disorder by microarray analysis, wherein, the pathogenic role of DUSP1 in depression was further confirmed [48]. Finally, given that the pathological core of prion protein (PrP27–30) contains cofilin and Hsp90 [152], it is plausible that DUSP mediated regulation of cofilin, and potentially Hsp90, could contribute to the modulation of the pathogenesis of prion diseases. Therefore, DUSP manipulation could also offer therapeutic avenues in the aforementioned diseases.

The Clinical Trials website (https://clinicaltrials.gov/) as on 30 August 2017, lists trials for (a) the changes in DUSP1 expression in response to treatment for depression; (b) cross-sectional and longitudinal study of individuals with autism and germline heterozygous *PTEN* mutations; (c) everolimus drug and neurocognition in PTEN hamartoma tumor syndrome; and (d) DUSP6 expression changes as biomarker in response to non-small cell lung cancer, thyroid cancer, and advanced solid tumor treatments. Furthermore, there are a few compounds currently under investigation in clinical trials that are also DUSP-manipulating compounds, including magnesium chloride, arsenite, pentamidine, and PTP inhibitors. Those compounds are tested for their efficacy in various clinical conditions, such as cancer, arthritis, muscular dystrophy, seizures, depression, diabetic neuropathy, neuropathic pain, and infertility. It is worth noting that only DUSP23 and PTEN targeting drugs are listed under experimental status on the DrugBank online resource [153].

Mouse models carrying targeted manipulation of individual *DUSP* genes have been reported in literature. These in vivo models of DUSPs could be utilized for experimental works addressing how each DUSP members may play a role in the modulation of neuronal proteostasis as well as neurodegeneration. We provide a list of reported mouse models previously employed in biomedical studies in Table 2. Comprehensive information on transgenic or mutated strains is readily accessible from online resources, such as the Jackson Laboratory (https://www.jax.org/), the Knockout Mouse Project (KOMP; https://www.komp.org/), the Mutant Mouse Resource and Research Centers (MMRRC; https://www.mmrrc.org/), and the International Mouse Strain Resource (IMSR, http://www.findmice.org/). Additional modes of DUSP manipulation could include immunotherapies, gene therapies, and blood-based therapies. Currently, there is information available only with immunotherapies for PTP4A subfamily and gene therapy for PTEN in cancer treatments [148,154]. The potential advantages of modulating DUSPs may be evaluated in a case-by-case basis given the diversity of their subsequent downstream targets. Conditional manipulations of *DUSP* genes, instead of systemic manipulations, could be predicted to have a safer outcome and avoid potential embryonic lethality due to *DUSP* full knockout [155]. Together, these genetic tools will enable us to mechanistically address the critical roles of DUSPs in neuronal proteostasis.

## 8. Conclusions

With the present and emerging data, it is becoming more apparent that DUSPs are essential manipulators of neurotoxicity and neuronal proteostasis. We hope this review succeeds in providing a baseline upon which new studies can be founded.

## Figures and Tables

**Table 1:** The Correlation between Dysfunctional DUSP Members with Neural Abnormalities.

No.	Gene Name	Family & Domains	Possible Association with Neurological Deficits or Affected Neuronal Functions	Gene Expression in Indicative Brain Regions
1	*DUSP1*	a, b, c, d, e, Δ	HD [19]	CCx ^x^, CbCx ^x^, H ^x^, A ^y^, Sn ^y^
2	*DUSP2*	a, b, c, d, Δ	Seizure [31]	CCx ^x^, CbCx ^y^, H ^y^, A ^y^, Sn ^y^
3	*DUSP4*	a, b, c, d, Δ	Hippocampal synaptic function [32]	CCx ^y^, CbCx ^y^, H ^y^, A ^y^
4	*DUSP5*	a, b, c, d, Δ	Cerebral ischemia [33]	CCx ^y^, CbCx ^y^, H ^y^, A ^y^, SN ^y^, NAc ^y^
5	*DUSP6*	a, b, c, d, Δ	Glutamate-induced cytotoxicity [34]	CCx ^x^, CbCx ^x^, H ^x^, A ^y^, SN ^y^, NAc ^y^
6	*DUSP7*	a, b, c, d, Δ	ALS [35]	CCx ^y^, CbCx ^y^, H ^y^, A ^y^, SN ^y^, NAc ^y^
7	*DUSP8*	a, b, c, d, Δ	Cerebral ischemia [36]	CCx ^x^, CbCx ^x^, H ^x^, A ^y^, SN ^y^, NAc ^y^
8	*DUSP9*	a, b, c, d, Δ	Neural fate commitment [37]	H ^y^, A ^y^, NAc ^y^
9	*DUSP10*	a, b, c, d, e, Δ	Oligodendrocyte differentiation [38]	CCx ^x^, CbCx ^x^, H ^x^, A ^y^, SN ^y^, NAc ^y^
10	*DUSP16*	a, b, c, d, Δ	Axonal degeneration [39]	CCx ^x^, CbCx ^x^, H ^y^, A ^y^, SN ^y^, NAc ^y^
11	*STYXL1*	a, b, d, Δ	Neuronal differentiation [40]	CCx ^x^, CbCx ^x^, H ^x^, A ^y^, SN ^y^, NAc ^y^
12	*DUPD1*	a, b, e, Δ	Skeletal muscle atrophy [41]	CCx ^x^, CbCx ^x^, H ^x^
13	*DUSP3*	a, b, e, Δ	Glutamate-induced cytotoxicity [42]	CCx ^y^, CbCx ^y^, H ^y^, A ^y^, SN ^y^, NAc ^y^
14	*DUSP11*	a, b, Δ	Seizure [43]	CCx ^y^, CbCx ^y^, H ^y^, A ^y^, SN ^y^, NAc ^y^
15	*DUSP12*	a, b, f, Δ	Neuroblastoma GWAS [44]	CCx ^x^, CbCx ^y^, H ^y^, A ^y^, SN ^y^, NAc ^y^
16	*DUSP13*	a, b, e, Δ	Neuron development [45]	Some regions of CCx ^z^
17	*DUSP14*	a, b, e, Δ	HD [19]	CCx ^x^, CbCx ^x^, H ^x^, A ^y^, SN ^y^, NAc ^y^
18	*DUSP15*	a, b, e, g, Δ	Oligodendrocyte differentiation [46]	Low expression
19	*DUSP18*	a, b, e, Δ	SCI [47]	CCx ^x^, CbCx ^x^, H ^x^
20	*DUSP19*	a, b, e, Δ	Depression [48]	CCx ^x^
21	*DUSP21*	a, b, e, Δ	Not defined	Not defined
22	*DUSP22*	a, b, e, Δ	AD [49]	CCx ^x^, CbCx ^x^, H ^x^, A ^y^, SN ^y^, NAc ^y^
23	*DUSP23*	a, b, Δ	Neuronal differentiation [50]	CCx ^x^, CbCx ^y^, H ^x^, A ^y^, SN ^y^, NAc ^y^
24	*DUSP26*	a, b, e, Δ	AD [6]	CCx ^x^, CbCx ^x^, H ^x^, A ^y^, SN ^y^, NAc ^y^
25	*DUSP28*	a, b, Δ	Not defined	Low expression
26	*EPM2A*	a, b, h, i, j, Δ	Lafora disease [51]	CCx ^y^, CbCx ^y^, H ^y^, A ^y^, SN ^y^, NAc ^y^
27	*PTPMT1*	a, b, Δ	AD GWAS [52]	CCx ^x^, CbCx ^x^, H ^x^, A ^y^, SN ^y^, NAc ^y^
28	*RNGTT*	a, b, k, l, Δ	ASD RNA-Seq [53]	CCx ^x^, CbCx ^y^, H ^x^, A ^y^, SN ^y^, NAc ^y^
29	*STYX*	a, b, Δ	Golgi fragmentation [54]	CCx ^x^, CbCx ^x^, H ^x^, A ^y^, SN ^y^, NAc ^y^
30	*SSH1*	a, b, m, o, Δ	Synaptic plasticity [55]	CCx ^x^, CbCx ^y^, H ^x^, A ^y^, SN ^y^, NAc ^y^
31	*SSH2*	a, b, n, o, Δ	Neurite extension [56]	CCx ^x^, CbCx ^y^, H ^x^, A ^y^, SN ^y^, NAc ^y^
32	*SSH3*	a, b, n, o, Δ	Actin reorganization [57]	CCx ^x^, CbCx ^x^, H ^x^, A ^y^, SN ^y^, NAc ^y^
33	*PTP4A1*	a, b, Δ	Cerebral ischemia [58]	CCx ^y^, CbCx ^y^, H ^x^, A ^y^, SN ^y^, NAc ^y^
34	*PTP4A2*	a	NCL [59]	CCx ^y^, CbCx ^x^, H ^x^, A ^y^, SN ^y^, NAc ^y^
35	*PTP4A3*	a, b, Δ	MDD, Stress [60]	CCx ^y^, CbCx ^y^, H ^y^, A ^y^, SN ^y^, NAc ^y^
36	*CDC14A*	a, b, Δ	Diabetic stroke [61]	CCx ^x^, CbCx ^x^, H ^x^, A ^y^
37	*CDC14B*	a, b, Δ	Addictive behavior [62]	CCx ^x^, CbCx ^x^, H ^x^, A ^y^, SN ^y^, NA ^y^
38	*CDKN3*	a, p	Neuroblastoma [63]	Low expression
39	*PTPDC1*	a, b, Δ	PD GWAS [64]	CCx ^x^, CbCx ^x^, H ^x^, A ^y^, SN ^y^, NAc ^y^
40	*PTEN*	a, q, r, s, Δ	PD [65]	CCx ^x^, CbCx ^x^, H ^x^, A ^y^, SN ^y^, NAc ^y^
41	*TNS1*	a, r, s, t, u, v	Not defined	CCx ^x^, CbCx ^x^, H ^y^, A ^y^, SN ^y^, NAc ^y^
42	*TNS2*	a, r, s, t, u, v, w	Schizophrenia [66]	CCx ^y^, CbCx ^y^, H ^y^, A ^y^, SN ^y^, NAc ^y^
43	*TPTE*	a, r, s, Δ	Neuropathic pain [67]	Not defined
44	*TPTE2*	a, r, s, Δ	Not defined	Not defined

a: PTP-like; b: DUSP family; c: MKP subfamily; d: Rhodanese-like; e: Atypical DUSP subfamily; f: Zinc finger C2H2-type; g: SMAD/FHA; h: Immunoglobulin-like; i: Carbohydrate-binding; j: Laforin; k: mRNA capping enzyme; l: Nucleic-acid binding, OB fold; m: Protein phosphatase Slingshot Homolog 1; n: Protein phosphatase Slingshot; o: DEK, C-terminal; p: CDKN3; q: DUSP-PTEN; r: Tensin-type phosphatase; s: C2; t: SH2; u: PH domain-like; v: PTB/PI domain; w: Protein Kinase C-like/PE/DAG-binding; Δ: Dual-specificity phosphatase, catalytic domain; AD: Alzheimer’s disease; ALS: Amyotrophic lateral Sclerosis; ASD: Autism spectrum disorders; HD: Huntington’s disease; MDD: Major depressive disorder; NCL: Neuronal ceroid lipofuscinosis; PD: Parkinson’s disease; SCI: Spinal Cord Injury; GWAS: Genome-wide Association Studies; CCx: Cerebral cortex; CbCx: Cerebellar cortex; H: Hippocampus; A: Amygdala; SN: Substantia nigra; NAc: Nucleus accumbens; ^x^: Protein expression (data derived from the Human Protein Atlas [68], http://www.proteinatlas.org/); ^y^: RNA-seq data of Genotype-Tissue expression (GTEx) project (derived from the Expression Atlas at EMBL-EBI, [69], https://www.ebi.ac.uk/); ^z^: Microarray expression (derived from the © 2010 Allen Institute for Brain Science. Allen Human Brain Atlas. Available from: human.brain-map.org [70]).

**Table 2:** Modulation of Dual-Specificity Phosphatase by various modes.

S.No.	Gene Name	Inhibitors Validated in Biomedical Literature	Activators Validated in Biomedical Literature	Mouse Model Employed in Biomedical Literature *
1	*DUSP1*	BCI ^Φ^ [156], NSC 95397 ^Φ^ [157], NU-126 [158], Sanguinarine chloride ^Φ^ [159]	Salbutamol ^Φ^ [160], Formoretol ^Φ^ [160], Dexamethasone ^Φ^ [161], JWH015 ^Φ^ [162]	KO; Neuronal death [145]
2	*DUSP2*	Salubrinal ^Φ^ [163]	Not defined	KO; Arthritis [164]
3	*DUSP4*	Y [165]	Not defined	KO; Synaptic plasticity [32]
4	*DUSP5*	CSDDD2320, RR505, RR506, SM1842 [166]	Not defined	Transgenic; Inflammation [167]
5	*DUSP6*	BCI ^Φ^ [156], NSC 95397 ^Φ^ [157], NSC 45382 ^Φ^ [168], NSC 295642 ^Φ^ [168], NSC 357756 [168]	JWH015 ^Φ^ [162]	KO; Allodynia [169], Transgenic; FGFR signaling [170]
6	*DUSP7*	Y [171]	Not defined	Not defined
7	*DUSP8*	Arsenite ^Φ^, Anisomycin ^Φ^ inhibit the mouse ortholog M3/6 [172]	Not defined	KO, Transgenic; Ventricular remodeling [173]
8	*DUSP9*	Y [174,175]	Not defined	KO; Placental organogenesis [176]
9	*DUSP10*	AS077234-4 ^Φ^ [38]	Not defined	KO; Immune response [177]
10	*DUSP16*	Y [178]	Not defined	KO; Axon degeneration [39]
11	*STYXL1*	Not defined	Not defined	Not defined
12	*DUPD1*	NSC 95397 ^Φ^ [179], NSC 663284 ^Φ^ [179]	Not defined	Not defined
13	*DUSP3*	RK-682 ^Φ^ [180], MLS-0437605 [181], NU-126 [158], Isovenaciolide [182]	Not defined	KO; Angiogenesis [183]
14	*DUSP11*	Sodium (ortho)vanadate ^Φ^ [184], Magnesium Chloride ^Φ^ [184]	Not defined	KO; Immune response [185]
15	*DUSP12*	Zinc chelators (*Possibly)* [186]	Not defined	KO; Cardiac hypertrophy [187]
16	*DUSP13*	PTP inhibitor V ^Φ^ [188]	Not defined	Not defined
17	*DUSP14*	PTP inhibitor IV ^Φ^ [189], NSC-95397 ^Φ^ [190]	Not defined	KO; Immune response [191]
18	*DUSP15*	Y [192]	Not defined	Transgenic; Myelination [193]
19	*DUSP18*	Sodium orthovanadate ^Φ^ [194], Iodoaretic acid ^Φ^ [195]	Not defined	Not defined
20	*DUSP19*	Sodium (ortho)vanadate ^Φ^ [196]	Not defined	Not defined
21	*DUSP21*	Sodium orthovanadate ^Φ^ [194]	Not defined	Not defined
22	*DUSP22*	Sodium (ortho)vanadate ^Φ^ [196], BML-260 ^Φ^ [197], PRL-3 Inhibitor 1 ^Φ^ [198]	Not defined	KO; Immune response [199]
23	*DUSP23*	Sodium orthovanadate ^Φ^ [200], EDTA ^Φ^ [200], *N*-ethylmaleimide ^Φ^ [200], Y [201]	Not defined	Not defined
24	*DUSP26*	NSC-87877 ^Φ^ [202], Ethyl-3,4-dephostatin ^Φ^ [203], Y [204]	Not defined	Not defined
25	*DUSP28*	U0216 ^Φ^ [205]	Not defined	Not defined
26	*EPM2A*	Nitric oxide ^Φ^ [206], Glycogen ^Φ^ [207], polysaccharides ^Φ^ [207]	Not defined	KO; Lafora disease [208]
27	*PTPMT1*	Alexidine dihydrochloride ^Φ^ [209], Y [210]	Not defined	KO; Cardiolipin biosynthesis [155]
28	*RNGTT*	Mizoribine Monophosphate ^Φ^ [211]	Not defined	Not defined
29	*STYX*	Vandate (Sodium orthovanadate) ^Φ^ [212]	Not defined	Not defined
30	*SSH1*	Slingshot Inhibitor D3 ^Φ^ [213], Sennoside A ^Φ^ [214]	Not defined	Not defined
31	*SSH2*	Slingshot Inhibitor D3 ^Φ^ [213], Sennoside A ^Φ^ [214], ZINC04307500 [215]	Not defined	Not defined
32	*SSH3*	Sennoside A ^Φ^ [214]	Not defined	KO; Unknown [216]
33	*PTP4A1*	Thienopyridone ^Φ^ [217], Analog 3 ^Φ^ [218], Pentamidine ^Φ^ [219]	Not defined	CKO; Liver regeneration [220]
34	*PTP4A2*	Thienopyridone ^Φ^ [217], Analog 3 ^Φ^ [218], Pentamidine ^Φ^ [219]	Not defined	KO; Oncogenesis [221]
35	*PTP4A3*	BR-1 ^Φ^ [222], Analog 13 [223], PRL-3 inhibitor 1^Φ^ [224], Thienopyridone ^Φ^ [217], Analog 3 ^Φ^ [218], Pentamidine ^Φ^ [219]	Not defined	KO; Colon cancer [225]
36	*CDC14A*	Not defined	Not defined	Double KO; DDR [226]
37	*CDC14B*	Not defined	Not defined	CKO; DDR [227]
38	*CDKN3*	Sodium orthovanadate ^Φ^ [228]	Not defined	KO; Cancer [229]
39	*PTPDC1*	Not defined	Not defined	KO; Unknown [230]
40	*PTEN*	bpV(phen) ^Φ^ [231], bpV(pic) ^Φ^ [231], VO-Ohpic ^Φ^ [231], SF1670 ^Φ^ [231], bpV(HOpic) ^Φ^ [232]	Not defined	KO; Cortical dysplasia [233]
41	*TNS1*	Not defined	Not defined	KO; Angiogenesis [234]
42	*TNS2*	DHTS ^Φ^ [235]	Not defined	KO; Renal failure [236]
43	*TPTE*	Not defined	Not defined	Not defined
44	*TPTE2*	Not defined	Not defined	Not defined

^Φ^ Commercially available; Y: In silico predictions validated by enzyme assay; * Disease phenotype studied is indicated (not necessarily neurological); KO: Knock-out; CKO: Conditional Knock-out; FGFR: Fibroblast-growth factor receptor; DDR: DNA-damage response.

## Abbreviations

6-OHDA	6-Hydroxydopamine
AD	Alzheimer’s disease
ADF	Actin depolymerizing factor
ALS	Amyotrophic lateral sclerosis
ASK1	Apoptosis signal-regulating kinase 1
ATF	Activating transcription factor
ATG	Autophagy related
BCL	B-cell lymphoma
BIM	BCL-2 interacting mediator of cell death
CDC14	Cell division cycle 14
CDK	Cyclin-dependent kinase
CEBP/β	CCAAT/enhancer-binding protein β
CHOP	C/EBP homologous protein
DUSP	Dual-specificity phosphatase
eIF2α	Eukaryotic Initiation Factor 2 α
EGFR	Epidermal growth factor receptor
EPM2A	Epilepsy, Progressive Myoclonus type 2A (the gene encodes Laforin)
ERK	Extracellular signal–regulated kinase
HD	Huntington’s disease
HSF	Heat shock factor
Hsp	Heat shock protein
HSR	Heat shock response
IP3R	Inositol trisphosphate receptor
IRE1α	Inositol-requiring enzyme 1
JIP	JNK-interacting protein-1
JNK	c-Jun N-terminal kinase
LC3	Microtubule-associated protein 1A/1B-light chain 3
LIMK	Lin11, Isl-1 and Mec-3 domain kinase
MAPK	Mitogen-activated protein kinase
MK-2	MAPK-activated protein kinase 2
MKP	MAPK phosphatase
MOMP	Mitochondrial outer membrane permeabilization
mTORC1	Mammalian target of rapamycin complex 1
OxR	Oxidative stress
PD	Parkinson’s disease
PERK	Protein kinase RNA-like endoplasmic reticulum kinase
PI(5)P	Phosphatidylinositol 5-phosphate
PI3K/AKT	Phosphoinositide 3-kinase/Protein kinase B
PIP3	Phosphatidylinositol 3,4,5 trisphosphate
PN	Proteostasis network
PP2B	Protein phosphatase 2B
PTEN	Phosphatase and tensin homolog
PTP	Protein tyrosine phosphatase
PUMA	p53 Upregulated modulator of apoptosis
ROS	Reactive oxygen species
RYR	Ryanodine receptor
SSH	Slingshot protein phosphatase
STAT	Signal transducer and activator of transcription
STEP	Striatal-enriched protein tyrosine phosphatase
STYX(L1)	Serine/threonine/tyrosine-interacting-like protein
ULK	Unc-51 like autophagy activating kinase
VH1	Vaccinia virus H1 phosphatase

## Appendix A

**Table A1:** List of the Alternative Names of Dual Specificity Phosphatase (DUSP) Family Members.

No.	Gene Name	Entrez Gene ID *	UniProtKB ^§^	Alternative Name (s) *
1	*DUSP1*	1843	P28562	HVH1; MKP1; CL100; MKP-1; PTPN10
2	*DUSP2*	1844	Q05923	PAC-1
3	*DUSP4*	1846	Q13115	TYP; HVH2; MKP2; MKP-2
4	*DUSP5*	1847	Q16690	DUSP; HVH3
5	*DUSP6*	1848	Q16828	HH19; MKP3; PYST1
6	*DUSP7*	1849	Q16829	MKPX; PYST2
7	*DUSP8*	1850	Q13202	HB5; HVH8; HVH-5; C11orf81
8	*DUSP9*	1852	Q99956	MKP4; MKP-4
9	*DUSP10*	11221	Q9Y6W6	MKP5; MKP-5
10	*DUSP16*	80824	Q9BY84	MKP7; MKP-7
11	*STYXL1*	51657	Q9Y6J8	DUSP24; MKSTYX; MK-STYX
12	*DUPD1*	338599	Q68J44	FMDSP; DUSP27
13	*DUSP3*	1845	P51452	VHR
14	*DUSP11*	8446	O75319	PIR1
15	*DUSP12*	11266	Q9UNI6	YVH1; DUSP1
16	*DUSP13*	51207	Q9UII6	BEDP; MDSP; TMDP; SKRP4; DUSP13A; DUSP13B
17	*DUSP14*	11072	O95147	MKP6; MKP-L
18	*DUSP15*	128853	Q9H1R2	VHY; C20orf57
19	*DUSP18*	150290	Q8NEJ0	DSP18; DUSP20; LMWDSP20
20	*DUSP19*	142679	Q8WTR2	SKRP1; DUSP17; LMWDSP3; TS-DSP1
21	*DUSP21*	63904	Q9H596	LMWDSP21
22	*DUSP22*	56940	Q9NRW4	VHX; JKAP; JSP1; MKPX; JSP-1; MKP-x; LMWDSP2; LMW-DSP2
23	*DUSP23*	54935	Q9BVJ7	VHZ; MOSP; LDP-3; DUSP25
24	*DUSP26*	78986	Q9BV47	MKP8; NEAP; DSP-4; LDP-4; MKP-8; NATA1; SKRP3; DUSP24
25	*DUSP28*	285193	Q4G0W2	VHP; DUSP26
26	*EPM2A*	7957	O95278	EPM2; MELF
27	*PTPMT1*	114971	Q8WUK0	PLIP; 1110001D10Rik; 2810004N20Rik
28	*RNGTT*	8732	O60942	HCE; HCE1; hCAP; CAP1A
29	*STYX*	6815	Q8WUJ0	STYX
30	*SSH1*	54434	Q8WYL5	SSH1L
31	*SSH2*	85464	Q76I76	SSH-2; SSH-2L
32	*SSH3*	54961	Q8TE77	SSH3L
33	*PTP4A1*	7803	Q93096	HH72; PRL1; PRL-1; PTPCAAX1; PTP(CAAX1)
34	*PTP4A2*	8073	Q12974	HH13; OV-1; PRL2; HH7-2; PRL-2; PTP4A; HU-PP-1; PTPCAAX2; ptp-IV1a; ptp-IV1b
35	*PTP4A3*	11156	O75365	PRL3; PRL-3; PRL-R
36	*CDC14A*	8556	Q9UNH5	cdc14; hCDC14; DFNB105
37	*CDC14B*	8555	O60729	CDC14B3; Cdc14B1; Cdc14B2; hCDC14B
38	*CDKN3*	1033	Q16667	KAP; CDI1; CIP2; KAP1
39	*PTPDC1*	138639	A2A3K4	Naa-1; Ptpcd1; AI843923; AW456874
40	*PTEN*	5728	P60484	BZS; DEC; CWS1; GLM2; MHAM; TEP1; MMAC1; PTEN1; 10q23del; PTEN β
41	*TNS1*	7145	Q9HBL0	TNS; MXRA6; MST091; MST122; MST127; MSTP091; MSTP122; MSTP127; PPP1R155
42	*TNS2*	23371	Q63HR2	C1TEN; TENC1; C1-TEN
43	*TPTE*	7179	P56180	CT44; PTEN2
44	*TPTE2*	93492	Q6XPS3	TPIP

* Data obtained from Gene (Internet). Bethesda (MD): National Library of Medicine (US), National Center for Biotechnology Information (NCBI); 2004—[20170806]. Available from: https://www.ncbi.nlm.nih.gov/gene/ [237]; ^§^ Data obtained from UniProt [28], http://www.uniprot.org/.

## References

[1] Sala A.J., Bott L.C., Morimoto R.I. (2017). Shaping proteostasis at the cellular, tissue, and organismal level. J. Cell Biol..

[2] Wolff S., Weissman J.S., Dillin A. (2014). Differential scales of protein quality control. Cell.

[3] Labbadia J., Morimoto R.I. (2015). The biology of proteostasis in aging and disease. Annu. Rev. Biochem..

[4] Powers E.T., Balch W.E. (2013). Diversity in the origins of proteostasis networks—A driver for protein function in evolution. Nat. Rev. Mol. Cell Biol..

[5] Yerbury J.J., Ooi L., Dillin A., Saunders D.N., Hatters D.M., Beart P.M., Cashman N.R., Wilson M.R., Ecroyd H. (2016). Walking the tightrope: Proteostasis and neurodegenerative disease. J. Neurochem..

[6] Jung S., Nah J., Han J., Choi S.G., Kim H., Park J., Pyo H.K., Jung Y.K. (2016). Dual-specificity phosphatase 26 (dusp26) stimulates abeta42 generation by promoting amyloid precursor protein axonal transport during hypoxia. J. Neurochem..

[7] Calderwood S.K., Xie Y., Wang X., Khaleque M.A., Chou S.D., Murshid A., Prince T., Zhang Y. (2010). Signal transduction pathways leading to heat shock transcription. Signal Transduct. Insights.

[8] Darling N.J., Cook S.J. (2014). The role of mapk signalling pathways in the response to endoplasmic reticulum stress. Biochim. Biophys. Acta.

[9] Hutt D.M., Balch W.E. (2013). Expanding proteostasis by membrane trafficking networks. Cold Spring Harb. Perspect. Biol..

[10] Chen M.J., Dixon J.E., Manning G. (2017). Genomics and evolution of protein phosphatases. Sci. Signal..

[11] Tenreiro S., Eckermann K., Outeiro T.F. (2014). Protein phosphorylation in neurodegeneration: Friend or foe?. Front. Mol. Neurosci..

[12] Monteith W.B., Cohen R.D., Smith A.E., Guzman-Cisneros E., Pielak G.J. (2015). Quinary structure modulates protein stability in cells. Proc. Natl. Acad. Sci. USA.

[13] Alonso A., Rojas A., Godzik A., Mustelin T., Ariño J., Alexander D.R. (2004). The dual-specific protein tyrosine phosphatase family. Protein Phosphatases.

[14] Alonso A., Bayón Y. (2010). Atypical Dusps: 19 Phosphatases in Search of a Role.

[15] Mocciaro A., Schiebel E. (2010). Cdc14: A highly conserved family of phosphatases with non-conserved functions?. J. Cell Sci..

[16] Rios P., Li X., Kohn M. (2013). Molecular mechanisms of the prl phosphatases. Fed. Eur. Biochem. Soc. J..

[17] Haynie D.T. (2014). Molecular physiology of the tensin brotherhood of integrin adaptor proteins. Proteins.

[18] Collins L.M., Gavin A.M., Walsh S., Sullivan A.M., Wyatt S.L., O’Keeffe G.W., Nolan Y.M., Toulouse A. (2014). Expression of endogenous mkp1 in 6-ohda rat models of parkinson’s disease. Springerplus.

[19] Taylor D.M., Moser R., Regulier E., Breuillaud L., Dixon M., Beesen A.A., Elliston L., Silva Santos Mde F., Kim J., Jones L. (2013). Map kinase phosphatase 1 (mkp-1/dusp1) is neuroprotective in huntington’s disease via additive effects of jnk and p38 inhibition. J. Neurosci..

[20] Farooq A., Plotnikova O., Chaturvedi G., Yan S., Zeng L., Zhang Q., Zhou M.M. (2003). Solution structure of the mapk phosphatase pac-1 catalytic domain. Insights into substrate-induced enzymatic activation of mkp. Structure.

[21] Wu S., Vossius S., Rahmouni S., Miletic A.V., Vang T., Vazquez-Rodriguez J., Cerignoli F., Arimura Y., Williams S., Hayes T. (2009). Multidentate small-molecule inhibitors of vaccinia h1-related (vhr) phosphatase decrease proliferation of cervix cancer cells. J. Med. Chem..

[22] Jung S.K., Jeong D.G., Yoon T.S., Kim J.H., Ryu S.E., Kim S.J. (2007). Crystal structure of human slingshot phosphatase 2. Proteins.

[23] Jeong D.G., Kim S.J., Kim J.H., Son J.H., Park M.R., Lim S.M., Yoon T.S., Ryu S.E. (2005). Trimeric structure of prl-1 phosphatase reveals an active enzyme conformation and regulation mechanisms. J. Mol. Biol..

[24] Gray C.H., Good V.M., Tonks N.K., Barford D. (2003). The structure of the cell cycle protein cdc14 reveals a proline-directed protein phosphatase. EMBO J..

[25] Lee J.O., Yang H., Georgescu M.M., Di Cristofano A., Maehama T., Shi Y., Dixon J.E., Pandolfi P., Pavletich N.P. (1999). Crystal structure of the pten tumor suppressor: Implications for its phosphoinositide phosphatase activity and membrane association. Cell.

[26] Finn R.D., Attwood T.K., Babbitt P.C., Bateman A., Bork P., Bridge A.J., Chang H.-Y., Dosztányi Z., El-Gebali S., Fraser M. (2017). Interpro in 2017—Beyond protein family and domain annotations. Nucleic Acids Res..

[27] Tonks N.K. (2013). Protein tyrosine phosphatases—From housekeeping enzymes to master regulators of signal transduction. Fed. Eur. Biochem. Soc. J..

[28] (2017). Uniprot: The universal protein knowledgebase. Nucleic Acids Res..

[29] Sievers F., Wilm A., Dineen D., Gibson T.J., Karplus K., Li W., Lopez R., McWilliam H., Remmert M., Söding J. (2011). Fast, scalable generation of high-quality protein multiple sequence alignments using clustal omega. Mol. Syst. Biol..

[30] Goujon M., McWilliam H., Li W., Valentin F., Squizzato S., Paern J., Lopez R. (2010). A new bioinformatics analysis tools framework at EMBL–EBI. Nucleic Acids Res..

[31] Boschert U., Muda M., Camps M., Dickinson R., Arkinstall S. (1997). Induction of the dual specificity phosphatase pac1 in rat brain following seizure activity. NeuroReport.

[32] Abdul Rahman N.Z., Greenwood S.M., Brett R.R., Tossell K., Ungless M.A., Plevin R., Bushell T.J. (2016). Mitogen-activated protein kinase phosphatase-2 deletion impairs synaptic plasticity and hippocampal-dependent memory. J. Neurosci..

[33] Mengozzi M., Cervellini I., Villa P., Erbayraktar Z., Gokmen N., Yilmaz O., Erbayraktar S., Manohasandra M., Van Hummelen P., Vandenabeele P. (2012). Erythropoietin-induced changes in brain gene expression reveal induction of synaptic plasticity genes in experimental stroke. Proc. Natl. Acad. Sci. USA.

[34] Huang X., Liao W., Huang Y., Jiang M., Chen J., Wang M., Lin H., Guan S., Liu J. (2017). Neuroprotective effect of dual specificity phosphatase 6 against glutamate-induced cytotoxicity in mouse hippocampal neurons. Biomed. Pharmacother..

[35] Kudo L.C., Parfenova L., Vi N., Lau K., Pomakian J., Valdmanis P., Rouleau G.A., Vinters H.V., Wiedau-Pazos M., Karsten S.L. (2010). Integrative gene-tissue microarray-based approach for identification of human disease biomarkers: Application to amyotrophic lateral sclerosis. Hum. Mol. Genet..

[36] Huang Z., Liu Y., Zhu J., Wu H., Guo J. (2013). Involvement of the dual-specificity phosphatase m3/6 in c-jun n-terminal kinase inactivation following cerebral ischemia in the rat hippocampus. Int. J. Neurosci..

[37] Li Z., Fei T., Zhang J., Zhu G., Wang L., Lu D., Chi X., Teng Y., Hou N., Yang X. (2012). Bmp4 signaling acts via dual-specificity phosphatase 9 to control erk activity in mouse embryonic stem cells. Cell Stem Cell.

[38] Gobert R.P., Joubert L., Curchod M.L., Salvat C., Foucault I., Jorand-Lebrun C., Lamarine M., Peixoto H., Vignaud C., Fremaux C. (2009). Convergent functional genomics of oligodendrocyte differentiation identifies multiple autoinhibitory signaling circuits. Mol. Cell Biol..

[39] Maor-Nof M., Romi E., Sar Shalom H., Ulisse V., Raanan C., Nof A., Leshkowitz D., Lang R., Yaron A. (2016). Axonal degeneration is regulated by a transcriptional program that coordinates expression of pro- and anti-degenerative factors. Neuron.

[40] Flowers B.M., Rusnak L.E., Wong K.E., Banks D.A., Munyikwa M.R., McFarland A.G., Hinton S.D. (2014). The pseudophosphatase mk-styx induces neurite-like outgrowths in pc12 cells. PLoS ONE.

[41] West R., Waddell D. (2017). Dual specificity phosphatase and pro isomerase domain containing 1 (dupd1) is upregulated during neurogenic skeletal muscle atrophy and is differentially expressed in murf1-null mice. Fed. Am. Soc. Exp. Biol. J..

[42] Kim S.H., Markham J.A., Weiler I.J., Greenough W.T. (2008). Aberrant early-phase erk inactivation impedes neuronal function in fragile x syndrome. Proc. Natl. Acad. Sci. USA.

[43] Kedmi M., Orr-Urtreger A. (2007). Expression changes in mouse brains following nicotine-induced seizures: The modulation of transcription factor networks. Physiol. Genom..

[44] Tolbert V.P., Coggins G.E., Maris J.M. (2017). Genetic susceptibility to neuroblastoma. Curr. Opin. Genet. Dev..

[45] Park J.E., Park B.C., Kim H.A., Song M., Park S.G., Lee D.H., Kim H.J., Choi H.K., Kim J.T., Cho S. (2010). Positive regulation of apoptosis signal-regulating kinase 1 by dual-specificity phosphatase 13a. Cell Mol. Life Sci..

[46] Schmidt F., van den Eijnden M., Pescini Gobert R., Saborio G.P., Carboni S., Alliod C., Pouly S., Staugaitis S.M., Dutta R., Trapp B. (2012). Identification of vhy/dusp15 as a regulator of oligodendrocyte differentiation through a systematic genomics approach. PLoS ONE.

[47] Wen T., Hou J., Wang F., Zhang Y., Zhang T., Sun T. (2016). Comparative analysis of molecular mechanism of spinal cord injury with time based on bioinformatics data. Spinal Cord..

[48] Duric V., Banasr M., Licznerski P., Schmidt H.D., Stockmeier C.A., Simen A.A., Newton S.S., Duman R.S. (2010). A negative regulator of map kinase causes depressive behavior. Nat. Med..

[49] Sanchez-Mut J.V., Aso E., Heyn H., Matsuda T., Bock C., Ferrer I., Esteller M. (2014). Promoter hypermethylation of the phosphatase dusp22 mediates pka-dependent tau phosphorylation and creb activation in alzheimer’s disease. Hippocampus.

[50] Kim S.Y., Oh M., Lee K.S., Kim W.K., Oh K.J., Lee S.C., Bae K.H., Han B.S. (2016). Profiling analysis of protein tyrosine phosphatases during neuronal differentiation. Neurosci. Lett..

[51] Lynch D.S., Wood N.W., Houlden H. (2016). Late-onset lafora disease with prominent parkinsonism due to a rare mutation in epm2a. Neurol. Genet..

[52] Efthymiou A.G., Goate A.M. (2017). Late onset alzheimer’s disease genetics implicates microglial pathways in disease risk. Mol. Neurodegener..

[53] Ji X., Kember R.L., Brown C.D., Bucan M. (2016). Increased burden of deleterious variants in essential genes in autism spectrum disorder. Proc. Natl. Acad. Sci. USA.

[54] Dahal A., Hinton S.D. (2017). Antagonistic roles for styx pseudophosphatases in neurite outgrowth. Biochem. Soc. Trans..

[55] Yuen E.Y., Liu W., Kafri T., van Praag H., Yan Z. (2010). Regulation of ampa receptor channels and synaptic plasticity by cofilin phosphatase slingshot in cortical neurons. J. Physiol..

[56] Endo M., Ohashi K., Mizuno K. (2007). Lim kinase and slingshot are critical for neurite extension. J. Biol. Chem..

[57] Ohta Y., Kousaka K., Nagata-Ohashi K., Ohashi K., Muramoto A., Shima Y., Niwa R., Uemura T., Mizuno K. (2003). Differential activities, subcellular distribution and tissue expression patterns of three members of slingshot family phosphatases that dephosphorylate cofilin. Genes Cells.

[58] Takano S., Fukuyama H., Fukumoto M., Kimura J., Xue J.H., Ohashi H., Fujita J. (1996). Prl-1, a protein tyrosine phosphatase, is expressed in neurons and oligodendrocytes in the brain and induced in the cerebral cortex following transient forebrain ischemia. Brain Res. Mol. Brain Res..

[59] Von Schantz C., Saharinen J., Kopra O., Cooper J.D., Gentile M., Hovatta I., Peltonen L., Jalanko A. (2008). Brain gene expression profiles of cln1 and cln5 deficient mice unravels common molecular pathways underlying neuronal degeneration in ncl diseases. BMC Genom..

[60] Pajer K., Andrus B.M., Gardner W., Lourie A., Strange B., Campo J., Bridge J., Blizinsky K., Dennis K., Vedell P. (2012). Discovery of blood transcriptomic markers for depression in animal models and pilot validation in subjects with early-onset major depression. Transl. Psychiatry.

[61] Su J., Zhou H., Tao Y., Guo Z., Zhang S., Zhang Y., Huang Y., Tang Y., Hu R., Dong Q. (2015). Hcdc14a is involved in cell cycle regulation of human brain vascular endothelial cells following injury induced by high glucose, free fatty acids and hypoxia. Cell Signal..

[62] Wang J., Cui W., Wei J., Sun D., Gutala R., Gu J., Li M.D. (2011). Genome-wide expression analysis reveals diverse effects of acute nicotine exposure on neuronal function-related genes and pathways. Front. Psychiatry.

[63] Partridge V., Du L. (2017). The role of cdkn3 in neuroblastoma differentiation. Fed. Am. Soc. Exp. Biol. J..

[64] Hu Y., Deng L., Zhang J., Fang X., Mei P., Cao X., Lin J., Wei Y., Zhang X., Xu R. (2016). A pooling genome-wide association study combining a pathway analysis for typical sporadic Parkinson’s disease in the Han population of Chinese mainland. Mol. Neurobiol..

[65] Ihle N.T., Abraham R.T. (2017). The pten-parkin axis: At the nexus of cancer and neurodegeneration. Mol. Cell.

[66] Goudarzi S., Smith L.J., Schutz S., Hafizi S. (2013). Interaction of disc1 with the ptb domain of tensin2. Cell Mol. Life Sci..

[67] Lu S., Ma S., Wang Y., Huang T., Zhu Z., Zhao G. (2017). Mus musculus-microrna-449a ameliorates neuropathic pain by decreasing the level of kcnma1 and trpa1, and increasing the level of tpte. Mol. Med. Rep..

[68] Uhlén M., Fagerberg L., Hallström B.M., Lindskog C., Oksvold P., Mardinoglu A., Sivertsson Å., Kampf C., Sjöstedt E., Asplund A. (2015). Tissue-based map of the human proteome. Science.

[69] Petryszak R., Keays M., Tang Y.A., Fonseca N.A., Barrera E., Burdett T., Füllgrabe A., Fuentes A.M.-P., Jupp S., Koskinen S. (2016). Expression atlas update—An integrated database of gene and protein expression in humans, animals and plants. Nucleic Acids Res..

[70] Hawrylycz M.J., Lein E.S., Guillozet-Bongaarts A.L., Shen E.H., Ng L., Miller J.A., van de Lagemaat L.N., Smith K.A., Ebbert A., Riley Z.L. (2012). An anatomically comprehensive atlas of the adult human brain transcriptome. Nature.

[71] Patterson K.I., Brummer T., O’brien P.M., Daly R.J. (2009). Dual-specificity phosphatases: Critical regulators with diverse cellular targets. Biochem. J..

[72] Harper S.J., Wilkie N. (2003). Mapks: New targets for neurodegeneration. Expert Opin. Ther. Targets.

[73] Boutros T., Chevet E., Metrakos P. (2008). Mitogen-activated protein (map) kinase/map kinase phosphatase regulation: Roles in cell growth, death, and cancer. Pharmacol. Rev..

[74] Colucci-D’Amato L., Perrone-Capano C., di Porzio U. (2003). Chronic activation of erk and neurodegenerative diseases. Bioessays.

[75] Cruz C.D., Cruz F. (2007). The erk 1 and 2 pathway in the nervous system: From basic aspects to possible clinical applications in pain and visceral dysfunction. Curr. Neuropharmacol..

[76] Haeusgen W., Boehm R., Zhao Y., Herdegen T., Waetzig V. (2009). Specific activities of individual c-jun n-terminal kinases in the brain. Neuroscience.

[77] Takeda K., Ichijo H. (2002). Neuronal p38 mapk signalling: An emerging regulator of cell fate and function in the nervous system. Genes Cells.

[78] Jeffrey K.L., Camps M., Rommel C., Mackay C.R. (2007). Targeting dual-specificity phosphatases: Manipulating map kinase signalling and immune responses. Nat. Rev. Drug Discov..

[79] Kumar S., Wirths O., Stuber K., Wunderlich P., Koch P., Theil S., Rezaei-Ghaleh N., Zweckstetter M., Bayer T.A., Brustle O. (2016). Phosphorylation of the amyloid beta-peptide at ser26 stabilizes oligomeric assembly and increases neurotoxicity. Acta Neuropathol..

[80] Sambataro F., Pennuto M. (2017). Post-translational modifications and protein quality control in motor neuron and polyglutamine diseases. Front. Mol. Neurosci..

[81] Braithwaite S.P., Stock J.B., Lombroso P.J., Nairn A.C. (2012). Protein phosphatases and Alzheimer’s disease. Prog. Mol. Biol. Transl. Sci..

[82] Das I., Krzyzosiak A., Schneider K., Wrabetz L., D’Antonio M., Barry N., Sigurdardottir A., Bertolotti A. (2015). Preventing proteostasis diseases by selective inhibition of a phosphatase regulatory subunit. Science.

[83] Jeanneteau F., Deinhardt K., Miyoshi G., Bennett A.M., Chao M.V. (2010). The map kinase phosphatase mkp-1 regulates bdnf-induced axon branching. Nat. Neurosci..

[84] Monteiro F.A., Sousa M.M., Cardoso I., do Amaral J.B., Guimaraes A., Saraiva M.J. (2006). Activation of erk1/2 map kinases in familial amyloidotic polyneuropathy. J. Neurochem..

[85] Banzhaf-Strathmann J., Benito E., May S., Arzberger T., Tahirovic S., Kretzschmar H., Fischer A., Edbauer D. (2014). Microrna-125b induces tau hyperphosphorylation and cognitive deficits in alzheimer’s disease. EMBO J..

[86] Willoughby E.A., Collins M.K. (2005). Dynamic interaction between the dual specificity phosphatase mkp7 and the jnk3 scaffold protein beta-arrestin 2. J. Biol. Chem..

[87] Thathiah A., Horre K., Snellinx A., Vandewyer E., Huang Y., Ciesielska M., De Kloe G., Munck S., De Strooper B. (2013). Beta-arrestin 2 regulates abeta generation and gamma-secretase activity in alzheimer’s disease. Nat. Med..

[88] Checler F., Dunys J., Pardossi-Piquard R., Alves da Costa C. (2010). P53 is regulated by and regulates members of the gamma-secretase complex. Neurodegener. Dis..

[89] Lokareddy R.K., Bhardwaj A., Cingolani G. (2013). Atomic structure of dual-specificity phosphatase 26, a novel p53 phosphatase. Biochemistry.

[90] Sekine Y., Tsuji S., Ikeda O., Sato N., Aoki N., Aoyama K., Sugiyama K., Matsuda T. (2006). Regulation of stat3-mediated signaling by lmw-dsp2. Oncogene.

[91] Ohashi K. (2015). Roles of cofilin in development and its mechanisms of regulation. Dev. Growth Differ..

[92] Bamburg J.R., Bernstein B.W., Davis R.C., Flynn K.C., Goldsbury C., Jensen J.R., Maloney M.T., Marsden I.T., Minamide L.S., Pak C.W. (2010). Adf/cofilin-actin rods in neurodegenerative diseases. Curr. Alzheimer Res..

[93] Niwa R., Nagata-Ohashi K., Takeichi M., Mizuno K., Uemura T. (2002). Control of actin reorganization by slingshot, a family of phosphatases that dephosphorylate adf/cofilin. Cell.

[94] Zafar S., Younas N., Sheikh N., Tahir W., Shafiq M., Schmitz M., Ferrer I., Andréoletti O., Zerr I. (2017). Cytoskeleton-associated risk modifiers involved in early and rapid progression of sporadic creutzfeldt-jakob disease. Mol. Neurobiol..

[95] Kreis P., Leondaritis G., Lieberam I., Eickholt B.J. (2014). Subcellular targeting and dynamic regulation of pten: Implications for neuronal cells and neurological disorders. Front. Mol. Neurosci..

[96] Cui W., Wang S., Wang Z., Wang Z., Sun C., Zhang Y. (2017). Inhibition of pten attenuates endoplasmic reticulum stress and apoptosis via activation of pi3k/akt pathway in alzheimer’s disease. Neurochem. Res..

[97] Zhang X., Li F., Bulloj A., Zhang Y.W., Tong G., Zhang Z., Liao F.F., Xu H. (2006). Tumor-suppressor pten affects tau phosphorylation, aggregation, and binding to microtubules. Fed. Am. Soc. Exp. Biol. J..

[98] Benarroch E.E. (2011). Heat shock proteins: Multiple neuroprotective functions and implications for neurologic disease. Neurology.

[99] Stetler R.A., Gan Y., Zhang W., Liou A.K., Gao Y., Cao G., Chen J. (2010). Heat shock proteins: Cellular and molecular mechanisms in the central nervous system. Prog. Neurobiol..

[100] Bakthisaran R., Tangirala R., Rao Ch M. (2015). Small heat shock proteins: Role in cellular functions and pathology. Biochim. Biophys. Acta.

[101] Smith H.L., Li W., Cheetham M.E. (2015). Molecular chaperones and neuronal proteostasis. Semin. Cell Dev. Biol..

[102] Yaglom J., O’Callaghan-Sunol C., Gabai V., Sherman M.Y. (2003). Inactivation of dual-specificity phosphatases is involved in the regulation of extracellular signal-regulated kinases by heat shock and hsp72. Mol. Cell Biol..

[103] Satoh J., Tabira T., Yamamura T., Kim S.U. (1994). Hsp72 induction by heat stress is not universal in mammalian neural cell lines. J. Neurosci. Res..

[104] Palacios C., Collins M.K., Perkins G.R. (2001). The jnk phosphatase m3/6 is inhibited by protein-damaging stress. Curr. Biol..

[105] Merienne K., Helmlinger D., Perkin G.R., Devys D., Trottier Y. (2003). Polyglutamine expansion induces a protein-damaging stress connecting heat shock protein 70 to the jnk pathway. J. Biol. Chem..

[106] Song H., Kim W., Kim S.H., Kim K.T. (2016). Vrk3-mediated nuclear localization of hsp70 prevents glutamate excitotoxicity-induced apoptosis and abeta accumulation via enhancement of erk phosphatase vhr activity. Sci. Rep..

[107] Hu Y., Mivechi N.F. (2006). Association and regulation of heat shock transcription factor 4b with both extracellular signal-regulated kinase mitogen-activated protein kinase and dual-specificity tyrosine phosphatase dusp26. Mol. Cell Biol..

[108] Sharda P.R., Bonham C.A., Mucaki E.J., Butt Z., Vacratsis P.O. (2009). The dual-specificity phosphatase hyvh1 interacts with hsp70 and prevents heat-shock-induced cell death. Biochem. J..

[109] Woodford M.R., Truman A.W., Dunn D.M., Jensen S.M., Cotran R., Bullard R., Abouelleil M., Beebe K., Wolfgeher D., Wierzbicki S. (2016). Mps1 mediated phosphorylation of hsp90 confers renal cell carcinoma sensitivity and selectivity to hsp90 inhibitors. Cell Rep..

[110] Kondoh K., Nishida E. (2007). Regulation of map kinases by map kinase phosphatases. Biochim. Biophys. Acta.

[111] Simard J.P., Reynolds D.N., Kraguljac A.P., Smith G.S., Mosser D.D. (2011). Overexpression of hsp70 inhibits cofilin phosphorylation and promotes lymphocyte migration in heat-stressed cells. J. Cell Sci..

[112] Birben E., Sahiner U.M., Sackesen C., Erzurum S., Kalayci O. (2012). Oxidative stress and antioxidant defense. World Allergy Organ. J..

[113] Manoharan S., Guillemin G.J., Abiramasundari R.S., Essa M.M., Akbar M., Akbar M.D. (2016). The role of reactive oxygen species in the pathogenesis of alzheimer’s disease, parkinson’s disease, and huntington’s disease: A mini review. Oxid. Med. Cell. Longev..

[114] Jeong D.G., Wei C.H., Ku B., Jeon T.J., Chien P.N., Kim J.K., Park S.Y., Hwang H.S., Ryu S.Y., Park H. (2014). The family-wide structure and function of human dual-specificity protein phosphatases. Acta Crystallogr. D Biol. Crystallogr..

[115] Chu C.T., Levinthal D.J., Kulich S.M., Chalovich E.M., DeFranco D.B. (2004). Oxidative neuronal injury. The dark side of erk1/2. Eur. J. Biochem..

[116] Kidger A.M., Keyse S.M. (2016). The regulation of oncogenic ras/erk signalling by dual-specificity mitogen activated protein kinase phosphatases (mkps). Semin. Cell Dev. Biol..

[117] Koga S., Kojima S., Kishimoto T., Kuwabara S., Yamaguchi A. (2012). Over-expression of map kinase phosphatase-1 (mkp-1) suppresses neuronal death through regulating jnk signaling in hypoxia/re-oxygenation. Brain Res..

[118] Martire S., Mosca L., d’Erme M. (2015). Parp-1 involvement in neurodegeneration: A focus on alzheimer’s and parkinson’s diseases. Mech. Ageing Dev..

[119] Hocsak E., Szabo V., Kalman N., Antus C., Cseh A., Sumegi K., Eros K., Hegedus Z., Gallyas F., Sumegi B. (2017). Parp inhibition protects mitochondria and reduces ros production via parp-1-atf4-mkp-1-mapk retrograde pathway. Free Radic. Biol. Med..

[120] Oehrl W., Cotsiki M., Panayotou G. (2013). Differential regulation of m3/6 (dusp8) signaling complexes in response to arsenite-induced oxidative stress. Cell Signal..

[121] Xu Q.Q., Xiao F.J., Sun H.Y., Shi X.F., Wang H., Yang Y.F., Li Y.X., Wang L.S., Ge R.L. (2016). Ptpmt1 induced by hif-2alpha regulates the proliferation and glucose metabolism in erythroleukemia cells. Biochem. Biophys. Res. Commun..

[122] Karch C.M., Ezerskiy L.A., Bertelsen S., Goate A.M. (2016). Alzheimer’s disease risk polymorphisms regulate gene expression in the zcwpw1 and the celf1 loci. PLoS ONE.

[123] Liu X.Y., Zhang L.J., Chen Z., Liu L.B. (2017). The pten inhibitor bpv(pic) promotes neuroprotection against amyloid beta-peptide (25-35)-induced oxidative stress and neurotoxicity. Neurol. Res..

[124] Yu L., Kelly U., Ebright J.N., Malek G., Saloupis P., Rickman D.W., McKay B.S., Arshavsky V.Y., Bowes Rickman C. (2007). Oxidative stress-induced expression and modulation of phosphatase of regenerating liver-1 (prl-1) in mammalian retina. Biochim. Biophys. Acta.

[125] Karkali K., Panayotou G. (2012). The *Drosophila* dusp puckered is phosphorylated by jnk and p38 in response to arsenite-induced oxidative stress. Biochem. Biophys. Res. Commun..

[126] Kim J.S., Huang T.Y., Bokoch G.M. (2009). Reactive oxygen species regulate a slingshot-cofilin activation pathway. Mol. Biol. Cell.

[127] Hetz C., Mollereau B. (2014). Disturbance of endoplasmic reticulum proteostasis in neurodegenerative diseases. Nat. Rev. Neurosci..

[128] Ron D., Walter P. (2007). Signal integration in the endoplasmic reticulum unfolded protein response. Nat. Rev. Mol. Cell Biol..

[129] Rininger A., Dejesus C., Totten A., Wayland A., Halterman M.W. (2012). Mkp-1 antagonizes c/ebpbeta activity and lowers the apoptotic threshold after ischemic injury. Cell Death Differ..

[130] Zhang W., Neo S.P., Gunaratne J., Poulsen A., Boping L., Ong E.H., Sangthongpitag K., Pendharkar V., Hill J., Cohen S.M. (2015). Feedback regulation on pten/akt pathway by the er stress kinase perk mediated by interaction with the vault complex. Cell Signal..

[131] Kim K.H., Lee M.S. (2014). Autophagy—A key player in cellular and body metabolism. Nat. Rev. Endocrinol..

[132] Esclatine A., Chaumorcel M., Codogno P. (2009). Macroautophagy signaling and regulation. Curr. Top. Microbiol. Immunol..

[133] Subramaniam S., Unsicker K. (2006). Extracellular signal-regulated kinase as an inducer of non-apoptotic neuronal death. Neuroscience.

[134] Zhou Y.Y., Li Y., Jiang W.Q., Zhou L.F. (2015). Mapk/jnk signalling: A potential autophagy regulation pathway. Biosci. Rep..

[135] Sui X., Kong N., Ye L., Han W., Zhou J., Zhang Q., He C., Pan H. (2014). P38 and jnk mapk pathways control the balance of apoptosis and autophagy in response to chemotherapeutic agents. Cancer Lett..

[136] Nixon R.A. (2013). The role of autophagy in neurodegenerative disease. Nat. Med..

[137] Wang J., Zhou J.Y., Kho D., Reiners J.J., Wu G.S. (2016). Role for dusp1 (dual-specificity protein phosphatase 1) in the regulation of autophagy. Autophagy.

[138] Fu M.-M., Nirschl J.J., Holzbaur E.L.F. (2014). Lc3 binding to the scaffolding protein jip1 regulates processive dynein-driven transport of autophagosomes. Dev. Cell.

[139] Yeasmin A.M., Waliullah T.M., Kondo A., Ushimaru T. (2015). Yvh1 protein phosphatase is required for pre-autophagosomal structure formation after torc1 inactivation. Biosci. Biotechnol. Biochem..

[140] Chen J.H., Zhang P., Chen W.D., Li D.D., Wu X.Q., Deng R., Jiao L., Li X., Ji J., Feng G.K. (2015). Atm-mediated pten phosphorylation promotes pten nuclear translocation and autophagy in response to DNA-damaging agents in cancer cells. Autophagy.

[141] Aguado C., Sarkar S., Korolchuk V.I., Criado O., Vernia S., Boya P., Sanz P., de Cordoba S.R., Knecht E., Rubinsztein D.C. (2010). Laforin, the most common protein mutated in lafora disease, regulates autophagy. Hum. Mol. Genet..

[142] Elmore S. (2007). Apoptosis: A review of programmed cell death. Toxicol. Pathol..

[143] Okouchi M., Ekshyyan O., Maracine M., Aw T.Y. (2007). Neuronal apoptosis in neurodegeneration. Antioxid. Redox Signal..

[144] Wada T., Penninger J.M. (2004). Mitogen-activated protein kinases in apoptosis regulation. Oncogene.

[145] Kristiansen M., Hughes R., Patel P., Jacques T.S., Clark A.R., Ham J. (2010). Mkp1 is a c-jun target gene that antagonizes jnk-dependent apoptosis in sympathetic neurons. J. Neurosci..

[146] Niemi N.M., Lanning N.J., Klomp J.A., Tait S.W., Xu Y., Dykema K.J., Murphy L.O., Gaither L.A., Xu H.E., Furge K.A. (2011). Mk-styx, a catalytically inactive phosphatase regulating mitochondrially dependent apoptosis. Mol. Cell Biol..

[147] Huyer G., Liu S., Kelly J., Moffat J., Payette P., Kennedy B., Tsaprailis G., Gresser M.J., Ramachandran C. (1997). Mechanism of inhibition of protein-tyrosine phosphatases by vanadate and pervanadate. J. Biol. Chem..

[148] Rios P., Nunes-Xavier C.E., Tabernero L., Kohn M., Pulido R. (2014). Dual-specificity phosphatases as molecular targets for inhibition in human disease. Antioxid. Redox Signal..

[149] Lee D.H., Cho S. (2014). Dual-specificity phosphatase 8 promotes the degradation of the polyglutamine protein ataxin-1. Bull. Korean Chem. Soc..

[150] Zhang Z., Pinto A.M., Wan L., Wang W., Berg M.G., Oliva I., Singh L.N., Dengler C., Wei Z., Dreyfuss G. (2013). Dysregulation of synaptogenesis genes antecedes motor neuron pathology in spinal muscular atrophy. Proc. Natl. Acad. Sci. USA.

[151] Isrie M., Zamani Esteki M., Peeters H., Voet T., Van Houdt J., Van Paesschen W., Van Esch H. (2015). Homozygous missense mutation in styxl1 associated with moderate intellectual disability, epilepsy and behavioural complexities. Eur. J. Med. Genet..

[152] Giorgi A., Di Francesco L., Principe S., Mignogna G., Sennels L., Mancone C., Alonzi T., Sbriccoli M., De Pascalis A., Rappsilber J. (2009). Proteomic profiling of prp27-30-enriched preparations extracted from the brain of hamsters with experimental scrapie. Proteomics.

[153] Wishart D.S., Knox C., Guo A.C., Shrivastava S., Hassanali M., Stothard P., Chang Z., Woolsey J. (2006). Drugbank: A comprehensive resource for in silico drug discovery and exploration. Nucleic Acids Res..

[154] Dillon L.M., Miller T.W. (2014). Therapeutic targeting of cancers with loss of pten function. Curr. Drug Targets.

[155] Zhang J., Guan Z., Murphy A.N., Wiley S.E., Perkins G.A., Worby C.A., Engel J.L., Heacock P., Nguyen O.K., Wang J.H. (2011). Mitochondrial phosphatase ptpmt1 is essential for cardiolipin biosynthesis. Cell Metab..

[156] Molina G., Vogt A., Bakan A., Dai W., Queiroz de Oliveira P., Znosko W., Smithgall T.E., Bahar I., Lazo J.S., Day B.W. (2009). Zebrafish chemical screening reveals an inhibitor of dusp6 that expands cardiac cell lineages. Nat. Chem. Biol..

[157] Vogt A., McDonald P.R., Tamewitz A., Sikorski R.P., Wipf P., Skoko J.J., Lazo J.S. (2008). A cell-active inhibitor of mitogen-activated protein kinase phosphatases restores paclitaxel-induced apoptosis in dexamethasone-protected cancer cells. Mol. Cancer Ther..

[158] Lazo J.S., Nunes R., Skoko J.J., de Oliveira P.E.Q., Vogt A., Wipf P. (2006). Novel benzofuran inhibitors of human mitogen-activated protein kinase phosphatase-1. Bioorg. Med. Chem..

[159] Vogt A., Tamewitz A., Skoko J., Sikorski R.P., Giuliano K.A., Lazo J.S. (2005). The benzo[c]phenanthridine alkaloid, sanguinarine, is a selective, cell-active inhibitor of mitogen-activated protein kinase phosphatase-1. J. Biol. Chem..

[160] Alkhouri H., Rumzhum N.N., Rahman M.M., FitzPatrick M., de Pedro M., Oliver B.G., Bourke J.E., Ammit A.J. (2014). Tlr2 activation causes tachyphylaxis to beta2-agonists in vitro and ex vivo: Modelling bacterial exacerbation. Allergy.

[161] Manetsch M., Ramsay E.E., King E.M., Seidel P., Che W., Ge Q., Hibbs D.E., Newton R., Ammit A.J. (2012). Corticosteroids and beta(2)-agonists upregulate mitogen-activated protein kinase phosphatase 1: In vitro mechanisms. Br. J. Pharmacol..

[162] Landry R.P., Martinez E., DeLeo J.A., Romero-Sandoval E.A. (2012). Spinal cannabinoid receptor type 2 agonist reduces mechanical allodynia and induces mitogen-activated protein kinase phosphatases in a rat model of neuropathic pain. J. Pain.

[163] Hamamura K., Nishimura A., Chen A., Takigawa S., Sudo A., Yokota H. (2015). Salubrinal acts as a dusp2 inhibitor and suppresses inflammation in anti-collagen antibody-induced arthritis. Cell Signal..

[164] Jeffrey K.L., Brummer T., Rolph M.S., Liu S.M., Callejas N.A., Grumont R.J., Gillieron C., Mackay F., Grey S., Camps M. (2006). Positive regulation of immune cell function and inflammatory responses by phosphatase pac-1. Nat. Immunol..

[165] Park H., Jeon T.J., Chien P.N., Park S.Y., Oh S.M., Kim S.J., Ryu S.E. (2014). Discovery of novel dusp4 inhibitors through the virtual screening with docking simulations. Bull. Korean Chem. Soc..

[166] Neumann T.S., Span E.A., Kalous K.S., Bongard R., Gastonguay A., Lepley M.A., Kutty R.G., Nayak J., Bohl C., Lange R.G. (2015). Identification of inhibitors that target dual-specificity phosphatase 5 provide new insights into the binding requirements for the two phosphate pockets. BMC Biochem..

[167] Kovanen P.E., Bernard J., Al-Shami A., Liu C., Bollenbacher-Reilley J., Young L., Pise-Masison C., Spolski R., Leonard W.J. (2008). T-cell development and function are modulated by dual specificity phosphatase dusp5. J. Biol. Chem..

[168] Vogt A., Cooley K.A., Brisson M., Tarpley M.G., Wipf P., Lazo J.S. (2003). Cell-active dual specificity phosphatase inhibitors identified by high-content screening. Chem. Biol..

[169] Saha M., Skopelja S., Martinez E., Alvarez D.L., Liponis B.S., Romero-Sandoval E.A. (2013). Spinal mitogen-activated protein kinase phosphatase-3 (mkp-3) is necessary for the normal resolution of mechanical allodynia in a mouse model of acute postoperative pain. J. Neurosci..

[170] Li C., Scott D.A., Hatch E., Tian X., Mansour S.L. (2007). Dusp6(mkp3) is a negative feedback regulator of fgf stimulated erk signaling during mouse development. Development.

[171] Park H.S., Jeon J.Y., Ryu S.E., Kim S.J. (2011). Discovery of novel inhibitors of dual-specificity phosphatase pyst2 with structure-based virtual screening. Bull. Korean Chem. Soc..

[172] Theodosiou A., Ashworth A. (2002). Differential effects of stress stimuli on a jnk-inactivating phosphatase. Oncogene.

[173] Liu R., van Berlo J.H., York A.J., Maillet M., Vagnozzi R.J., Molkentin J.D. (2016). Dusp8 regulates cardiac ventricular remodeling by altering erk1/2 signaling. Circ. Res..

[174] Park H., Jeon J.Y., Ryu S.E. (2012). Virtual screening and biochemical evaluation of mitogen-activated protein kinase phosphatase 4 inhibitors. Bull. Korean Chem. Soc..

[175] Ryu S.E., Kim S.J. (2014). Targeting allosteric sites for protein tyrosine phosphatase inhibition. Bio Des..

[176] Christie G.R., Williams D.J., MacIsaac F., Dickinson R.J., Rosewell I., Keyse S.M. (2005). The dual-specificity protein phosphatase dusp9/mkp-4 is essential for placental function but is not required for normal embryonic development. Mol. Cell Biol..

[177] Zhang Y., Blattman J.N., Kennedy N.J., Duong J., Nguyen T., Wang Y., Davis R.J., Greenberg P.D., Flavell R.A., Dong C. (2004). Regulation of innate and adaptive immune responses by map kinase phosphatase 5. Nature.

[178] Park H., Park S.Y., Nam S.-W., Ryu S.E. (2014). Discovery of novel dusp16 phosphatase inhibitors through virtual screening with homology modeled protein structure. J. Biomol. Screen..

[179] Devi Y.S., Seibold A.M., Shehu A., Maizels E., Halperin J., Le J., Binart N., Bao L., Gibori G. (2011). Inhibition of mapk by prolactin signaling through the short form of its receptor in the ovary and decidua: Involvement of a novel phosphatase. J. Biol. Chem..

[180] Hamaguchi T., Sudo T., Osada H. (1995). Rk-682, a potent inhibitor of tyrosine phosphatase, arrested the mammalian cell cycle progression at g1phase. Fed. Eur. Biochem. Soc. Lett..

[181] Musumeci L., Kuijpers M.J., Gilio K., Hego A., Theatre E., Maurissen L., Vandereyken M., Diogo C.V., Lecut C., Guilmain W. (2015). Dual-specificity phosphatase 3 deficiency or inhibition limits platelet activation and arterial thrombosis. Circulation.

[182] Ueda K., Usui T., Nakayama H., Ueki M., Takio K., Ubukata M., Osada H. (2002). 4-isoavenaciolide covalently binds and inhibits vhr, a dual-specificity phosphatase. Fed. Eur. Biochem. Soc. Lett..

[183] Amand M., Erpicum C., Bajou K., Cerignoli F., Blacher S., Martin M., Dequiedt F., Drion P., Singh P., Zurashvili T. (2014). Dusp3/vhr is a pro-angiogenic atypical dual-specificity phosphatase. Mol. Cancer.

[184] Deshpande T., Takagi T., Hao L., Buratowski S., Charbonneau H. (1999). Human pir1 of the protein-tyrosine phosphatase superfamily has RNA 5′-triphosphatase and diphosphatase activities. J. Biol. Chem..

[185] Nallaparaju K.C., Zhang Y., Liu X., Reynolds J.M., Nurieva R.I., Dong C. (2013). Dusp11 is a critical regulator of innate immune responses mediated by dendritic cells. Cytokine.

[186] Oteiza P.I. (2012). Zinc and the modulation of redox homeostasis. Free Radic. Biol. Med..

[187] Tilley D.G., Sabri A. (2017). Dusps as critical regulators of cardiac hypertrophy. Clin. Sci..

[188] Youn D., Cho S. (2013). Inhibition of dusp13a activity by ptp inhibitor v. Bull. Korean Chem. Soc..

[189] Park J.E., Park B.C., Song M., Park S.G., Lee D.H., Park S.Y., Kim J.H., Cho S. (2009). Ptp inhibitor iv protects jnk kinase activity by inhibiting dual-specificity phosphatase 14 (dusp14). Biochem. Biophys. Res. Commun..

[190] Seo H., Cho S. (2011). Specific inhibition of dusp14 by NSC-95397 in vitro. Bull. Korean Chem. Soc..

[191] Yang C.-Y., Li J.-P., Chiu L.-L., Lan J.-L., Chen D.-Y., Chuang H.-C., Huang C.-Y., Tan T.-H. (2014). Dual-specificity phosphatase 14 (dusp14/mkp6) negatively regulates tcr signaling by inhibiting tab1 activation. J. Immunol..

[192] Park H., Lee H.S., Kim S.J. (2015). Virtual screening with docking simulations and biochemical evaluation of vhy phosphatase inhibitors. Chem. Pharm. Bull..

[193] Muth K.N., Piefke S., Weider M., Sock E., Hermans-Borgmeyer I., Wegner M., Küspert M. (2016). The dual-specificity phosphatase dusp15 is regulated by sox10 and myrf in myelinating oligodendrocytes. Glia.

[194] Hood K.L., Tobin J.F., Yoon C. (2002). Identification and characterization of two novel low-molecular-weight dual specificity phosphatases. Biochem. Biophys. Res. Commun..

[195] Wu Q., Gu S., Dai J., Dai J., Wang L., Li Y., Zeng L., Xu J., Ye X., Zhao W. (2003). Molecular cloning and characterization of a novel dual-specificity phosphatase18 gene from human fetal brain. Biochim. Biophys. Acta Gene Struct. Expr..

[196] Zama T., Aoki R., Kamimoto T., Inoue K., Ikeda Y., Hagiwara M. (2002). A novel dual specificity phosphatase skrp1 interacts with the mapk kinase mkk7 and inactivates the jnk mapk pathway: Implication for the precise regulation of the particular mapk pathway. J. Biol. Chem..

[197] Doman T.N., McGovern S.L., Witherbee B.J., Kasten T.P., Kurumbail R., Stallings W.C., Connolly D.T., Shoichet B.K. (2002). Molecular docking and high-throughput screening for novel inhibitors of protein tyrosine phosphatase-1b. J. Med. Chem..

[198] Ju A.N., Cho S.Y. (2012). Inhibition of dual-specificity phosphatase 22 (dusp22) by prl-3 inhibitor i. Bull. Korean Chem. Soc..

[199] Li J.-P., Yang C.-Y., Chuang H.-C., Lan J.-L., Chen D.-Y., Chen Y.-M., Wang X., Chen A.J., Belmont J.W., Tan T.-H. (2014). The phosphatase jkap/dusp22 inhibits T-cell receptor signalling and autoimmunity by inactivating lck. Nat. Commun..

[200] Wu Q., Li Y., Gu S., Li N., Zheng D., Li D., Zheng Z., Ji C., Xie Y., Mao Y. (2004). Molecular cloning and characterization of a novel dual-specificity phosphatase 23 gene from human fetal brain. Int. J. Biochem. Cell Biol..

[201] Park H., Park S.Y., Oh J.J., Ryu S.E. (2013). Identification of potent vhz phosphatase inhibitors with structure-based virtual screening. J. Biomol. Screen..

[202] Shi Y., Ma I.T., Patel R.H., Shang X., Chen Z., Zhao Y., Cheng J., Fan Y., Rojas Y., Barbieri E. (2015). Nsc-87877 inhibits dusp26 function in neuroblastoma resulting in p53-mediated apoptosis. Cell Death Dis..

[203] Seo H., Cho S. (2016). Inhibition of dual-specificity phosphatase 26 by ethyl-3,4-dephostatin: Ethyl-3,4-dephostatin as a multiphosphatase inhibitor. Die Pharm. Int. J. Pharm. Sci..

[204] Park H., Kyung A., Lee H.J., Kang S., Yoon T.S., Ryu S.E., Jeong D.G. (2013). Virtual screening and biochemical evaluation of the inhibitors of dual-specificity phosphatase 26. Med. Chem. Res..

[205] Lee J., Hun Yun J., Lee J., Choi C., Hoon Kim J. (2015). Blockade of dual-specificity phosphatase 28 decreases chemo-resistance and migration in human pancreatic cancer cells. Sci. Rep..

[206] Toyota R., Honjo Y., Imajo R., Satoh A. (2016). *S*-nitrosylation of laforin inhibits its phosphatase activity and is implicated in lafora disease. Matters.

[207] Wang W., Roach P.J. (2004). Glycogen and related polysaccharides inhibit the laforin dual-specificity protein phosphatase. Biochem. Biophys. Res. Commun..

[208] Ganesh S., Delgado-Escueta A.V., Sakamoto T., Avila M.R., Machado-Salas J., Hoshii Y., Akagi T., Gomi H., Suzuki T., Amano K. (2002). Targeted disruption of the epm2a gene causes formation of lafora inclusion bodies, neurodegeneration, ataxia, myoclonus epilepsy and impaired behavioral response in mice. Hum. Mol. Genet..

[209] Doughty-Shenton D., Joseph J.D., Zhang J., Pagliarini D.J., Kim Y., Lu D., Dixon J.E., Casey P.J. (2010). Pharmacological targeting of the mitochondrial phosphatase ptpmt1. J. Pharmacol. Exp. Ther..

[210] Park H., Kim S.Y., Kyung A., Yoon T.S., Ryu S.E., Jeong D.G. (2012). Structure-based virtual screening approach to the discovery of novel ptpmt1 phosphatase inhibitors. Bioorg. Med. Chem. Lett..

[211] Picard-Jean F., Bougie I., Shuto S., Bisaillon M. (2013). The immunosuppressive agent mizoribine monophosphate is an inhibitor of the human RNA capping enzyme. PLoS ONE.

[212] Wishart M.J., Denu J.M., Williams J.A., Dixon J.E. (1995). A single mutation converts a novel phosphotyrosine binding domain into a dual-specificity phosphatase. J. Biol. Chem..

[213] Li K.-S., Xiao P., Zhang D.-L., Hou X.-B., Ge L., Yang D.-X., Liu H.-D., He D.-F., Chen X., Han K.-R. (2015). Identification of para-substituted benzoic acid derivatives as potent inhibitors of the protein phosphatase slingshot. Chem. Med. Chem..

[214] Lee S.Y., Kim W., Lee Y.G., Kang H.J., Lee S.H., Park S.Y., Min J.K., Lee S.R., Chung S.J. (2017). Identification of sennoside a as a novel inhibitor of the slingshot (ssh) family proteins related to cancer metastasis. Pharmacol. Res..

[215] Mui M.K.-H. (2011). Identification of Specific Inhibitors for a Dual-Specificity Phosphatase SSH-2.

[216] Kousaka K., Kiyonari H., Oshima N., Nagafuchi A., Shima Y., Chisaka O., Uemura T. (2008). Slingshot-3 dephosphorylates adf/cofilin but is dispensable for mouse development. Genesis.

[217] Daouti S., Li W.-H., Qian H., Huang K.-S., Holmgren J., Levin W., Reik L., McGady D.L., Gillespie P., Perrotta A. (2008). A selective phosphatase of regenerating liver phosphatase inhibitor suppresses tumor cell anchorage-independent growth by a novel mechanism involving p130cas cleavage. Cancer Res..

[218] Hoeger B., Diether M., Ballester P.J., Kohn M. (2014). Biochemical evaluation of virtual screening methods reveals a cell-active inhibitor of the cancer-promoting phosphatases of regenerating liver. Eur. J. Med. Chem..

[219] Pathak M.K., Dhawan D., Lindner D.J., Borden E.C., Farver C., Yi T. (2002). Pentamidine is an inhibitor of prl phosphatases with anticancer activity 1 supported in part by nih grants r01ca79891 and r01mg58893 (to T.Y.) and ca90914 (to E.C.B.). Mol. Cancer Ther..

[220] Jiao Y., Ye D.Z., Li Z., Teta-Bissett M., Peng Y., Taub R., Greenbaum L.E., Kaestner K.H. (2015). Protein tyrosine phosphatase of liver regeneration-1 is required for normal timing of cell cycle progression during liver regeneration. Am. J. Physiol. Gastrointest. Liver Physiol..

[221] Hardy S., Uetani N., Wong N., Kostantin E., Labbe D.P., Begin L.R., Mes-Masson A., Miranda-Saavedra D., Tremblay M.L. (2015). The protein tyrosine phosphatase prl-2 interacts with the magnesium transporter cnnm3 to promote oncogenesis. Oncogene.

[222] Zimmerman M.W., McQueeney K.E., Isenberg J.S., Pitt B.R., Wasserloos K.A., Homanics G.E., Lazo J.S. (2014). Protein-tyrosine phosphatase 4a3 (ptp4a3) promotes vascular endothelial growth factor signaling and enables endothelial cell motility. J. Biol. Chem..

[223] Salamoun J.M., McQueeney K.E., Patil K., Geib S.J., Sharlow E.R., Lazo J.S., Wipf P. (2016). Photooxygenation of an amino-thienopyridone yields a more potent ptp4a3 inhibitor. Org. Biomol. Chem..

[224] Ahn J.H., Kim S.J., Park W.S., Cho S.Y., Ha J.D., Kim S.S., Kang S.K., Jeong D.G., Jung S.K., Lee S.H. (2006). Synthesis and biological evaluation of rhodanine derivatives as prl-3 inhibitors. Bioorg. Med. Chem. Lett..

[225] Zimmerman M.W., Homanics G.E., Lazo J.S. (2013). Targeted deletion of the metastasis-associated phosphatase ptp4a3 (prl-3) suppresses murine colon cancer. PLoS ONE.

[226] Lin H., Ha K., Lu G., Fang X., Cheng R., Zuo Q., Zhang P. (2015). Cdc14a and cdc14b redundantly regulate DNA double-strand break repair. Mol. Cell. Biol..

[227] Wei Z., Peddibhotla S., Lin H., Fang X., Li M., Rosen J.M., Zhang P. (2011). Early-onset aging and defective DNA damage response in cdc14b-deficient mice. Mol. Cell. Biol..

[228] Hannon G.J., Casso D., Beach D. (1994). Kap: A dual specificity phosphatase that interacts with cyclin-dependent kinases. Proc. Natl. Acad. Sci. USA.

[229] Sun Z., Cerabona D., He Y., Nalepa G. (2016). Cdkn3 knockout mice develop hematopoietic malignancies. Blood.

[230] Takasuga A., Sato K., Nakamura R., Saito Y., Sasaki S., Tsuji T., Suzuki A., Kobayashi H., Matsuhashi T., Setoguchi K. (2015). Non-synonymous fgd3 variant as positional candidate for disproportional tall stature accounting for a carcass weight qtl (cw-3) and skeletal dysplasia in japanese black cattle. PLoS Genet..

[231] Spinelli L., Lindsay Y.E., Leslie N.R. (2015). Pten inhibitors: An evaluation of current compounds. Adv. Biol. Regul..

[232] Schmid A.C., Byrne R.D., Vilar R., Woscholski R. (2004). Bisperoxovanadium compounds are potent pten inhibitors. Fed. Eur. Biochem. Soc. Lett..

[233] Ljungberg M.C., Sunnen C.N., Lugo J.N., Anderson A.E., D’Arcangelo G. (2009). Rapamycin suppresses seizures and neuronal hypertrophy in a mouse model of cortical dysplasia. Dis. Models Mech..

[234] Shih Y.-P., Sun P., Wang A., Lo S.H. (2015). Tensin1 positively regulates rhoa activity through its interaction with dlc1. Biochim. Biophys. Acta.

[235] Ryu S.H., Lee J., Jeong H., Koh A. (2016). Pharmaceutical Compositions for Preventing or Treating Diabetic Nephropathy Comprising the Activity Inhibitor of Tenc1. U.S. Patent.

[236] Sasaki H., Marusugi K., Kimura J., Kitamura H., Nagasaki K.-I., Torigoe D., Agui T., Sasaki N. (2015). Genetic background-dependent diversity in renal failure caused by the tensin2 gene deficiency in the mouse. Biomed. Res..

[237] Coordinators N.R. (2016). Database resources of the national center for biotechnology information. Nucleic Acids Res..

